# Morphological Characteristics, Anatomical Structure, and Gene Expression: Novel Insights into Cytokinin Accumulation during Carrot Growth and Development

**DOI:** 10.1371/journal.pone.0134166

**Published:** 2015-07-28

**Authors:** Guang-Long Wang, Sheng Sun, Guo-Ming Xing, Xue-Jun Wu, Feng Wang, Ai-Sheng Xiong

**Affiliations:** 1 State Key Laboratory of Crop Genetics and Germplasm Enhancement, College of Horticulture, Nanjing Agricultural University, Nanjing, 210095, China; 2 College of Horticulture, Shanxi Agricultural University, Taigu, 030801, China; Saint Mary's University, CANADA

## Abstract

Cytokinins have been implicated in normal plant growth and development. These bioactive molecules are essential for cell production and expansion in higher plants. Carrot is an Apiaceae vegetable with great value and undergoes significant size changes over the process of plant growth. However, cytokinin accumulation and its potential roles in carrot growth have not been elucidated. To address this problem, carrot plants at five stages were collected, and morphological and anatomical characteristics and expression profiles of cytokinin-related genes were determined. During carrot growth and development, cytokinin levels were the highest at the second stage in the roots, whereas relatively stable levels were observed in the petioles and leaves. *DcCYP735A2* showed high expression at stage 2 in the roots, which may contribute largely to the higher cytokinin level at this stage. However, expression of most metabolic genes did not follow a pattern similar to that of cytokinin accumulation, indicating that cytokinin biosynthesis was regulated through a complex network. Genes involved in cytokinin signal perception and transduction were also integrated to normal plant growth and development. The results from the present work suggested that cytokinins may regulate plant growth in a stage-dependent manner. Our work would shed novel insights into cytokinin accumulation and its potential roles during carrot growth. Further studies regarding carrot cytokinins may be achieved by modification of the genes involved in cytokinin biosynthesis, inactivation, and perception.

## Introduction

Hormones are intrinsic plant growth regulators that act in response to environmental cues [1,2]. Among them, cytokinins (CKs) are a group of phytohormones that are involved in various aspects of plant growth, including reproductive development, seed germination, leaf senescence, photomorphogenesis, and meristem activity [3–5]. Naturally occurring CKs include isoprenoid CKs and aromatic CKs, although whether aromatic CKs are ubiquitous in all plants remains unclear [6].

**Fig 1:**
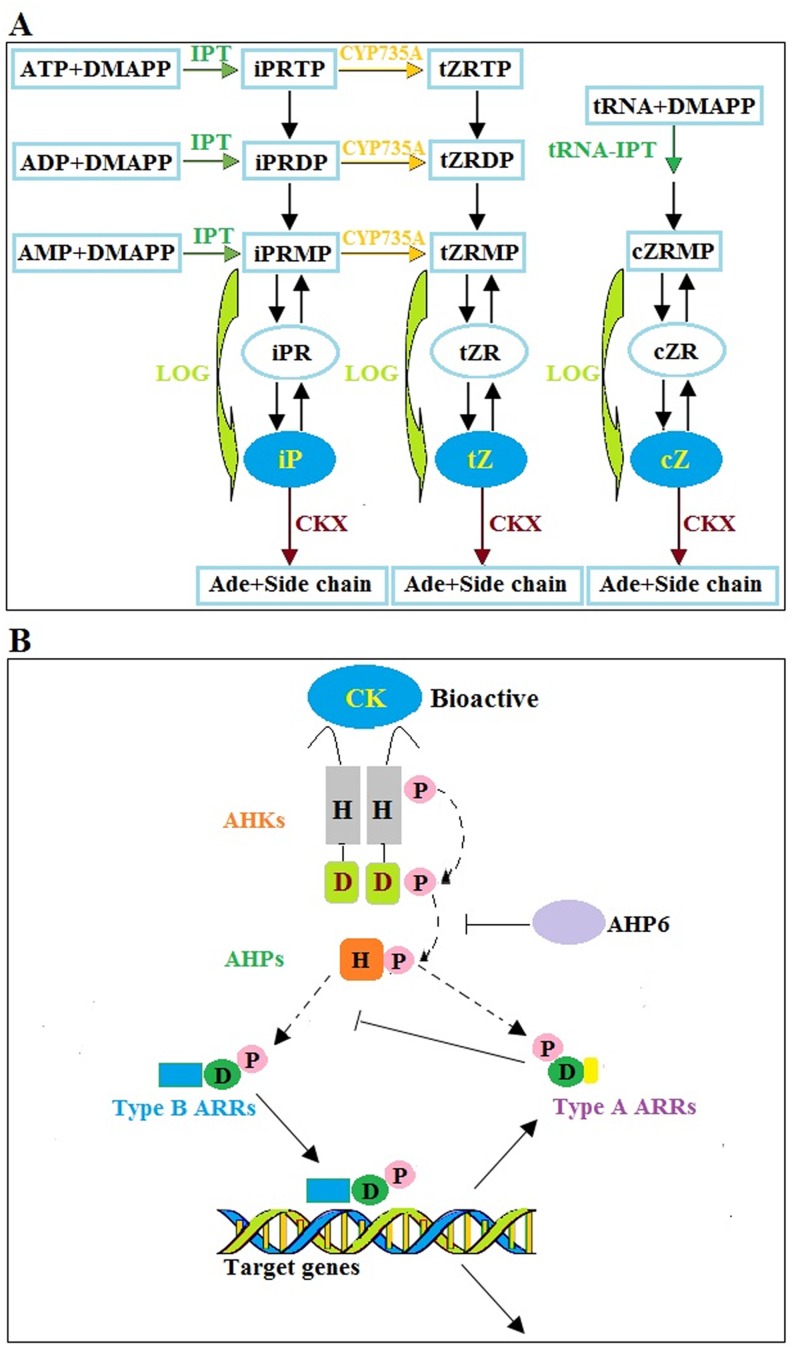
Proposed module of GA biosynthesis, inactivation, and perception in *Arabidopsis*.

The CK biosynthesis, degradation, and signaling pathways have been extensively studied in *Arabidopsis* [7,8] (Fig 1). Biosynthesis of iP-CKs (iP) and tZ-CKs (tZ) is initiated by a rate-limiting step, which is catalyzed by isopentenyl transferases (IPTs) [9,10]. Consequently, iP-nucleotides (iPRTP, iPRDP, and iPRMP) are converted to the corresponding tZ-nucleotides (tZRTP, tZRDP, and tZRMP) by cytochrome P450 monooxygenases (CYP735As) [11]. The conversion of active free bases is catalyzed by CK nucleoside 5′-monophosphate phosphoribohydrolases (LOGs) using monophosphates (iPRMP or tZRMP) as substrates [12]. iP and tZ are recognized as the most active CKs in most plants, including *Arabidopsis*. However, in some plant species, such as maize or rice, *cis*-zeatin CKs (cZ) that utilize tRNAs as prenyl acceptors are the major CK metabolites [13]. These bioactive CKs can be inactivated by CK dehydrogenases (CKXs) [14] (Fig 1A). In recent years, the CK signaling pathway has been proposed based on the identification of *Arabidopsis* mutants [15]. As previously described, CK conveys information through a complex two-component system [16]. In this model, CK binds to a CHASE domain of histidine kinase receptors (AHKs) and triggers a phosphorelay [17]. First, autophosphorylation occurs within the AHK receptor. The phosphoryl group is then transferred to nuclear response regulators (ARRs) *via* five histidine phosphotransfer proteins (AHP1–AHP5) [18]. AHP6, a pseudo AHP, impairs phosphotransfer by competing with AHP1–AHP 5 [19]. Two types of ARRs, type-A and type-B, exist. The phosphorylated type-B ARRs can bind DNA and activate the transcription of CK-regulated targets, including the type-A ARRs, which act as negative regulators of CK signaling [20] (Fig 1B).

Numerous studies have indicated that plant growth can be controlled by regulating the CK-related genes [21–23]. Carrot (*Daucus carota* L.) is an Apiaceae plant with great value [24]. CarrotDB, a genomic and transcriptomic database has been well established [25,26]. Carrot development involves successive changes in organ size, metabolism, and physiological processes [27,28]. This process is essential for carrot production and quality and has been an area of increasing interest [29]. CK is necessary for cell division of early embryogenesis, and it is believed to play important roles in anthocyanin accumulation, senescence, and root enlargement in carrot [30–33]. However, CK biosynthesis, inactivation, and response during carrot growth and development remain unclear.

The present work aimed to investigate CK accumulation during carrot growth and development. Morphological characteristics, anatomical structure, and expression levels of genes involved in CK metabolism and signaling were analyzed and discussed to comprehensively elucidate CK roles in carrot growth and development. The results from our work would provide novel insights into CK-mediated plant growth.

## Materials and Methods

### Plant material

*D*. *carota* L. cv. ‘Kurodagosun’ was selected and cultivated in an artificial chamber at the Nanjing Agricultural University (32°04′N, 118°85′E). Plants were grown in a mixture of vermiculite and organic soil (1:1; v/v). The artificial environment was maintained at 25°C for 16 h with a light intensity of 300 μmol m^-2^s^-1^ during daytime followed by 18°C for 8 h in the dark. Carrot plants were sampled at 25 (stage 1), 42 (stage 2), 60 (stage 3), 75 (stage 4), and 90 (stage 5) days after sowing. The developmental stages were classified based on morphological characteristics and dates. For biochemical and molecular analyses, various tissues including roots, petioles, and leaves at different development stages were harvested, frozen, and stored at −80°C.

### Anatomical structure analysis

Samples were harvested at 25, 42, 60, 75, and 90 days after sowing for anatomical structure analysis. We cut fresh samples into small pieces and immediately stored them in phosphate buffer (pH 7.2) with 2.5% glutaraldehyde. For safranin O/fast green staining, the slices were first deparaffinized in xylene and dehydrated with ethanol. After dewaxing, we stained the samples with 1% safranin O for 2 h and rinsed off the excess stain with water. Subsequently, slices were dehydrated in a series of ethanol and counterstained with 0.5% fast green for 15 s. Afterward, we washed out excess stain with ethanol and sealed the samples with neutral balsam. The presence of lignin was considered when the tissue sections were stained with red staining under a light microscope and fiber tissues were stained with green staining.

### Assay of bioactive CK levels

Sample grinding was performed in a mortar with 10 mL of 80% methanol extraction solution containing 1 mM butylated hydroxytoluene. The mixture was incubated at 4°C for 4 h. Afterward, the samples were centrifuged at 3500 *g* for 10 min and the supernatants were passed through a C_18_ Sep-Pak cartridge (Waters, Milford, MA). Efflux was collected and dried with N_2_. Residues were dissolved in PBS solution containing 0.1% (v/v) Tween 20 and 0.1% (w/v) gelatin (pH 7.5). To determine endogenous levels of bioactive CKs, including iP and ZT, indirect enzyme-linked immunosorbent assay (ELISA) was carried out as previously described [34,35]. The quantification of bioactive CKs by ELISA was performed at the Phytohormones Research Institute, China Agricultural University, Beijing, China.

### Total RNA isolation and cDNA synthesis

The total RNAs of carrot roots, petioles, and leaves were extracted using an RNA extraction kit (Tiangen, Beijing, China) according to the manufacturer’s instructions. RNA quality was then assessed with a One-Drop spectrophotometer. Total RNA was incubated at 42°C for 2 min with gDNA Eraser (TaKaRa, Dalian, China) to remove genomic DNA contamination. cDNA synthesis was strictly carried out as described in PrimeScript RT reagent kit (TaKaRa, Dalian, China). Finally, cDNA was diluted 15-fold, and 2 μL of this diluted cDNA was used for quantitative real-time PCR (qRT-PCR) analysis.

### Gene expression analysis by qRT-PCR

To identify the genes involved in CK metabolism and perception, CK-related genes of *Arabidopsis* were aligned with the sequences in CarrotDB, a genomic and transcriptomic database for carrot (Lab of Apiaceae Plant Genetics and Germplasm Enhancement, http://apiaceae.njau.edu.cn/carrotdb/index.php) [26]. The gene sequences were listed in Figures A-U in S1 File. The primers of each gene were shown in Tables 1 and 2. qRT-PCR was carried out in a Bio-Rad IQ5 real-time PCR System (Bio-Rad, CA, USA) using TaKaRa SYBR Premix *Ex Taq* (TaKaRa, Dalian, China). Each PCR mixture comprised a total volume of 20 μL, which contained 10 μL of SYBR Premix *Ex Taq*, 7.4 μL of sterile deionized water, 2 μL of diluted cDNA strand, and 0.4 μL of each primer. PCR was conducted following the manufacturer’s specifications. The conditions were controlled as follows: 95°C for 30 s, followed by 40 cycles at 95°C for 5 s and 60°C for 30 s. The raw data were listed in Table A in S1 File. The relative expression levels were normalized against those of the carrot reference gene, *DcACTIN* [36]. Data from *DcIPT3* in carrot leaves at 60 days after sowing were selected as standards for gene expression analysis.

**Table 1:** Description of genes involved in cytokinin biosynthesis and inactivation.

Gene symbol	Molecular function	Homologous locus in *Arabidopsis*	Primer sequences (forward/reverse)
*DcIPT3*	Adenylate isopentenyltransferase	AT3G63110	GAATGGAATGGTAGATGAGGCAAGACA/ TCTCTAACTGGCGGCAGGCTAG
*DcIPT5*	Adenylate isopentenyltransferase	AT5G19040	TTTGGATGCGACCGAGGCTTT/ GCCGATAGTGCCGAATCTTCTTC
*DcIPT9*	tRNA isopentenyltransferase	AT5G20040	GTAGACTCGCACTTGAACTCGCTAA/ GGACAACGAAGGCTTGGCAGAA
*DcCYP735A1*	Cytokinin hydroxylase	AT5G38450	CTTGTCGGAGACGCACCTGATAA/ CAATGTGACGCTGATGATACCAATCG
*DcCYP735A2*	Cytokinin hydroxylase	AT1G67110	ATATGGAGGATGCGAACACAAGATGG/ TGTAGAAAGGTGAAAGCGAGAACGAAA
*DcLOG1*	Cytokinin riboside 5'-monophosphate phosphoribohydrolase	AT2G28305	CTATTATGATAGCCTGCTTGCCTTGTT/ AGCCTCTAATGATTGGTCCACTTCC
*DcLOG3*	Cytokinin riboside 5'-monophosphate phosphoribohydrolase	AT2G37210	CAGAGATGGCTAGGCACTCAGATG/ AAGAGTTGTAGTATCCGTCCACATTGA
*DcLOG8*	Cytokinin riboside 5'-monophosphate phosphoribohydrolase	AT5G11950	GATTGACTTGGTGTATGGTGGTGGTAG/ TTACATCTCCAACAGTCTCGCCAGA
*DcCYX1*	Cytokinin dehydrogenase	AT2G41510	GCCACATTACCACAAGCAAGAAGAGT/ TCCAGGAGCAAGAAGTGCCAGAG
*DcCYX7*	Cytokinin dehydrogenase	AT5G21482	TCTGTACTGTCTGGAGGTCGCATT/ CCGTTAGCTCTAGCTTGTTGTTCGT

**Table 2:** Description of genes related to cytokinin perception.

Gene symbol	Molecular function	Homologous locus in *Arabidopsis*	Primer sequences (forward/reverse)
*DcHK2*	Histidine kinase	AT5G35750	TGAAGTCTCCACAGTACCGTCCAA/ AGAAGCATACGCAGAATCCAGAGTC
*DcHK3*	Histidine kinase	AT1G27320	GATGCTAATTGGCGATCCTGGAAGAT/ ACAAGATGGACCGTTACGAAGATATGC
*DcHP1a*	Histidine-containing phosphotransfer protein	AT3G21510	AGCATTGGAGCACAAAGAGTTCAGA/ ATCCACCAGCAGCCACAAGTTG
*DcHP1b*	Histidine-containing phosphotransfer protein	AT3G21510	TTCGTGGCTTCTGCGATGAACA/ CTTCCACAATCTGCCGCTCCAA
*DcHP3*	Histidine-containing phosphotransfer protein	AT5G39340	CGATGCCTGTGTTCACCAATTCAAG/ CATCTCAAGCACCCTTCCAAGTTCT
*DcRR-B1*	Two-component response regulator	AT4G16110	CACCGCAGAGGAACCGATGATG/ AGCTCCACCGACCAGACTACAC
*DcRR-B2*	Two-component response regulator	AT4G16110	CAAGCAGCCCTAGTTCAACAAAGC/ GCATATTCCTGTGTCCATTCTCTTCCA
*DcRR-B3*	Two-component response regulator	AT1G67710	GAAGGCAACCAGCAGTTCAGGAG/ GGTCGAGATCAGGCAATGACAGATG
*DcRR-B4*	Two-component response regulator	AT2G25180	TTCAATACTGCTCAGATGCCTCCTAAC/ CCGCCTTGGTGTTCTCCTCTTG
*DcRR-A1*	Two-component response regulator	AT3G57040	GCAGAACAGGAGGAAGTATCATCACA/ TTCTCTTCGTGTTATTGCCGTCATCT
*DcRR-A2*	Two-component response regulator	AT3G57040	GATGTTTAGAGGAAGGAGCAGATGAGT/ TGCTTCTTGCTGTAATGACTGAATCAC

### Statistical analysis

Differences in CK accumulation and gene expression during carrot development were detected by Duncan’s multiple-range test at a 0.05 probability level.

## Results

### Plant growth analysis

**Fig 2:**
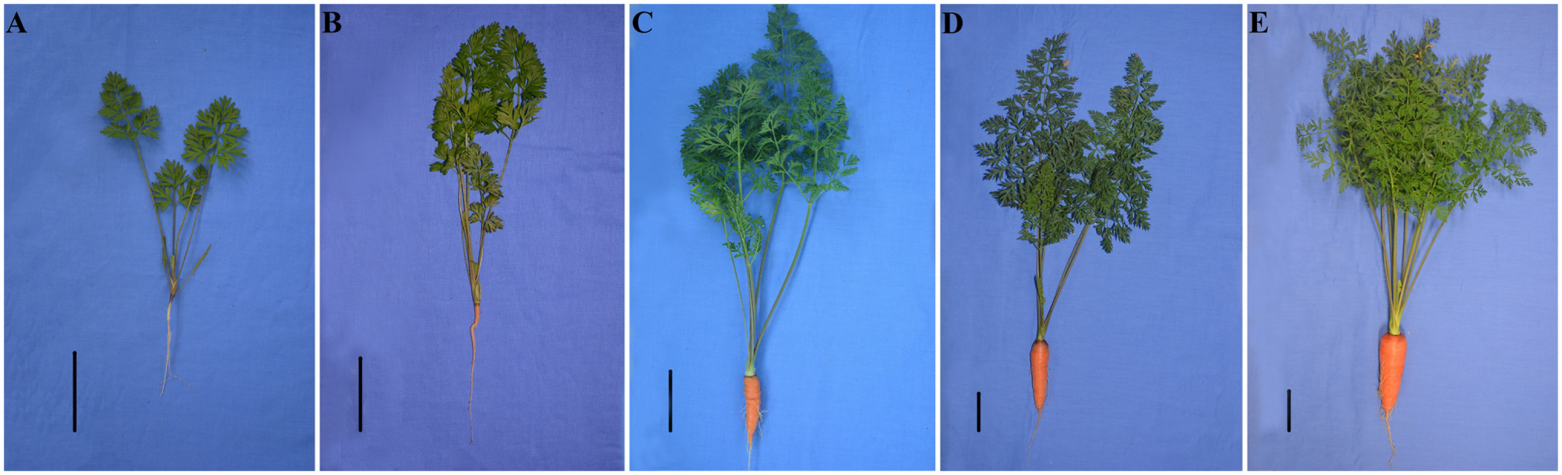
Growth status of carrots from five different developmental stages. A: Stage 1, 25-day-old. B: Stage 2, 40-day-old. C: Stage 3, 60-day-old. D: Stage 4, 75-day-old. E: Stage 5, 90-day-old. Black lines in the lower left corner of each plant represent 5 cm in that pixel.

**Fig 3:**
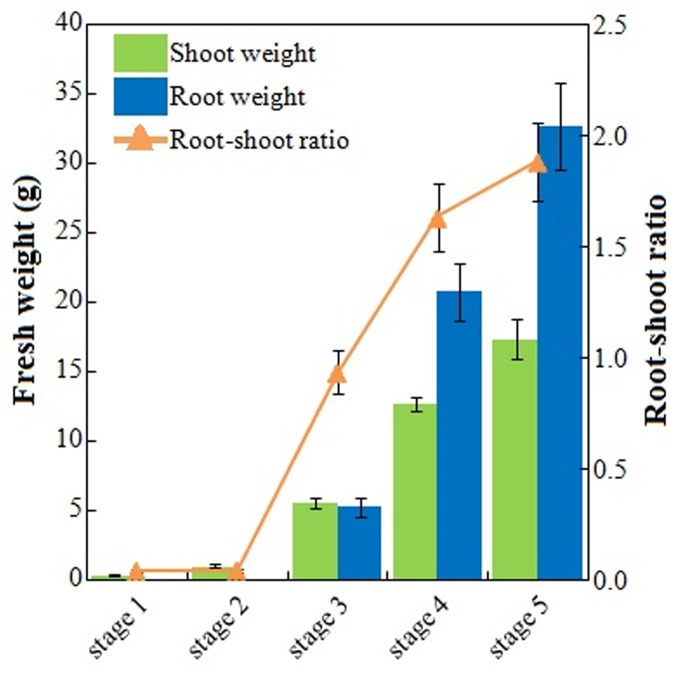
Characterization of root weight and shoot weight during carrot growth and development. Error bars represent standard deviation among three independent replicates. Data indicate mean ± SD of three replicates.

Roots, petioles, and leaves from 25-, 40-, 60-, 75-, and 90-day-old carrots were sampled (Fig 2). Root and shoot weights, along with root—shoot ratio, were measured to characterize plant growth during carrot development. The fresh weight of shoots was way above that of roots at stage 1. After 15 days, an orange color first appeared on the root surface. Shoot and root weights evidently increased at stage 2, whereas the root—shoot ratio was not significantly changed. The roots kept on enlarging, and root weight was approximately equal to shoot weight at the third stage. Finally, shoot weight was far less than that of root weight, and this trend was maintained at the final stage (Fig 3).

### Anatomical structure in the roots, petioles, and leaves

#### In the roots

**Fig 4:**
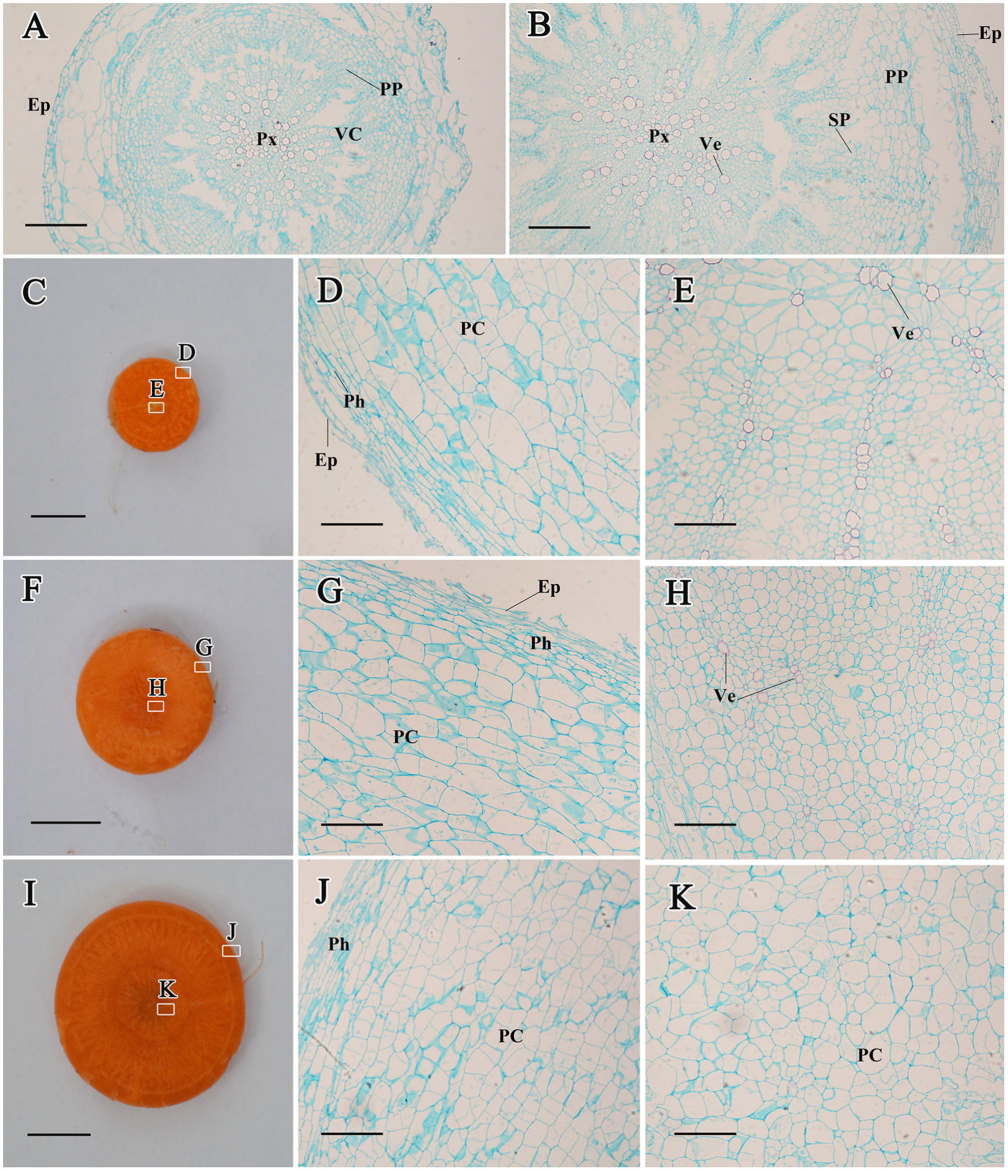
Structural changes in the carrot roots from stage 1 (A), stage 2 (B), stage 3 (C, D, and E), stage 4 (F, G, and H), and stage 5 (I, J, and K). Epidermis (Ep), parenchymalcell (PC), phellogen (Ph), primary phloem (PP), protoxylem (Px), secondary phloem (SP), vascularcambium (VC), and vessel (Ve) are marked in the Figure. Scale bars in A, B, D, E, G, H, J, and K are 100 μm in length, whereas scale bars in C, F, and I are 1 cm in length.

At stage 1, protoxylem (Px), vascularcambium (VC), primary phloem (PP), and epidermis were observed in the basic root structure, and no significant thickness was detected at this stage (Fig 4A). After 15 days, the secondary phloem (SP) region appeared between VC and PP (Fig 4B). Subsequently, parenchymalcells became larger, and the roots continued to enlarge (Fig 4C, 4F and 4I).

#### In the petioles

**Fig 5:**
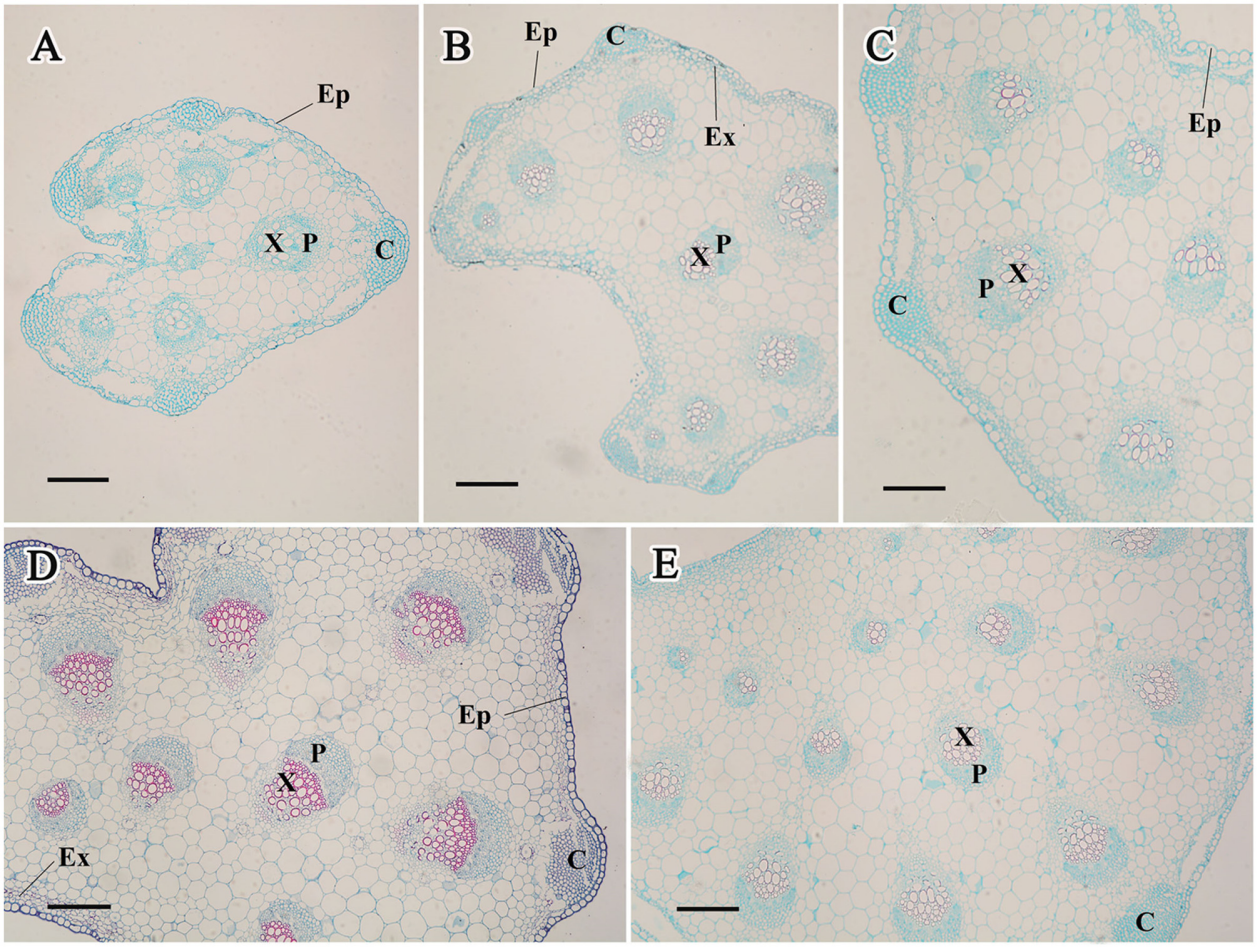
Structural changes in the carrot petioles from stage 1 (A), stage 2 (B), stage 3 (C), stage 4 (D), and stage 5 (E). Collenchyma (C), epidermis (Ep), exodermis (Ex), phloem (P), and xylem (X) are marked in the Figure. Scale bars in A, B, C, D, and E are 100 μm in length.

The petiole diameter increased over the process of carrot growth (Fig 5). Interestingly, the number of vascular bundles in the petioles also significantly increased, suggesting a constant thickness during this process.

#### In the leaves

**Fig 6:**
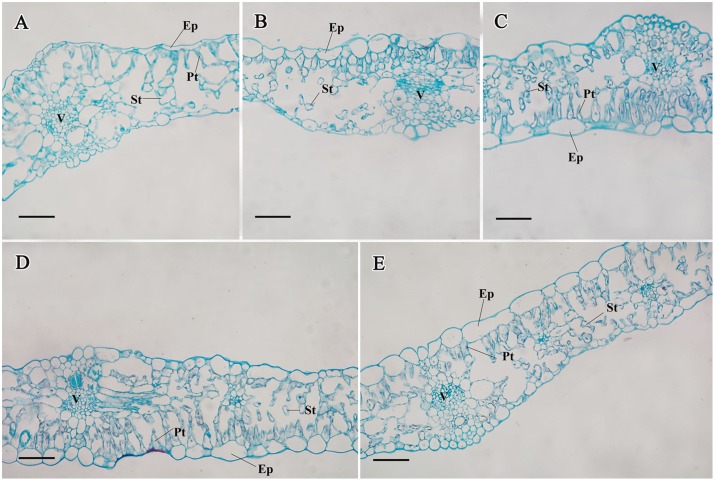
Structural changes in the carrot leaves from stage 1 (A), stage 2 (B), stage 3 (C), stage 4 (D), and stage 5 (E). Epidermis (Ep), palisade tissue (Pt), spongy tissue (St), and vascular tissue (V) are marked in the Figure. Scale bars in A, B, C, D, and E are 100 μm in length.

Palisade and spongy tissues are the main tissue types in mesophyll, which allow plant photosynthesis and gas exchange. At stages 1 and 2, the numbers of palisade and spongy cells were limited (Fig 6A and 6B). The palisade cells in the leaf obviously increased, and the ratio of palisade tissue to spongy tissue was high (Fig 6C, 6D and 6E).

### Changes in bioactive CKs

**Fig 7:**
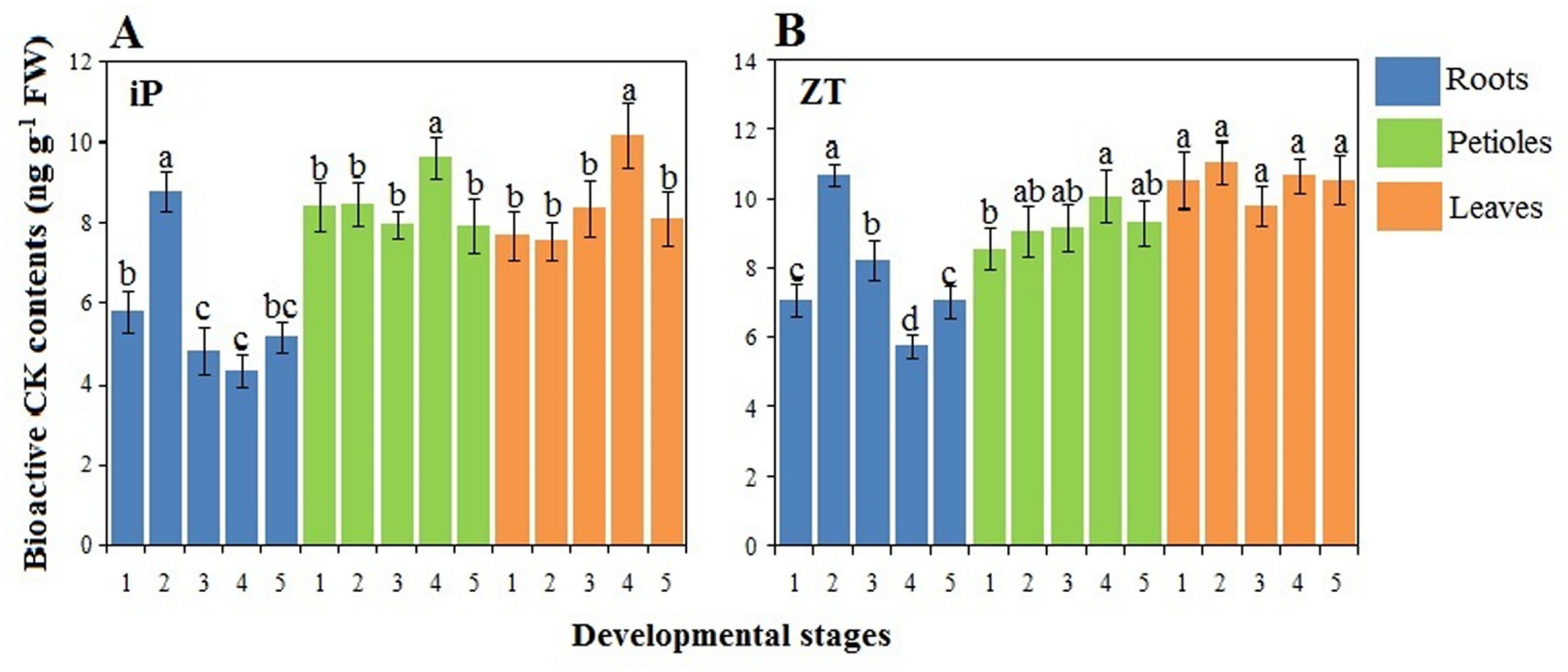
Bioactive CK levels in different tissues during carrot growth and development. Error bars represent standard deviation among three independent replicates. Data indicate mean ± SD of three replicates. Different lowercase letters indicate significant differences at *P* < 0.05.

The levels of bioactive CKs (iP and ZT) in roots, petioles, and leaves at different developmental stages were measured (Fig 7). In the roots, both iP and ZT levels were highest at stage 2 but suddenly decreased at stage 3. In the petioles, iP and ZT levels showed a similar pattern, which had the highest level at stage 4. The iP content in the leaves showed a trend similar to that in the petioles, whereas ZT was maintained at a relatively stable level during carrot growth. For each plant, both iP and ZT contents in the roots were lower than those in the petioles and leaves, except at stage 2. In addition, the ZT content during all stages was greater than the iP content.

### Expression profiles of genes involved in CK biosynthesis and inactivation

**Fig 8:**
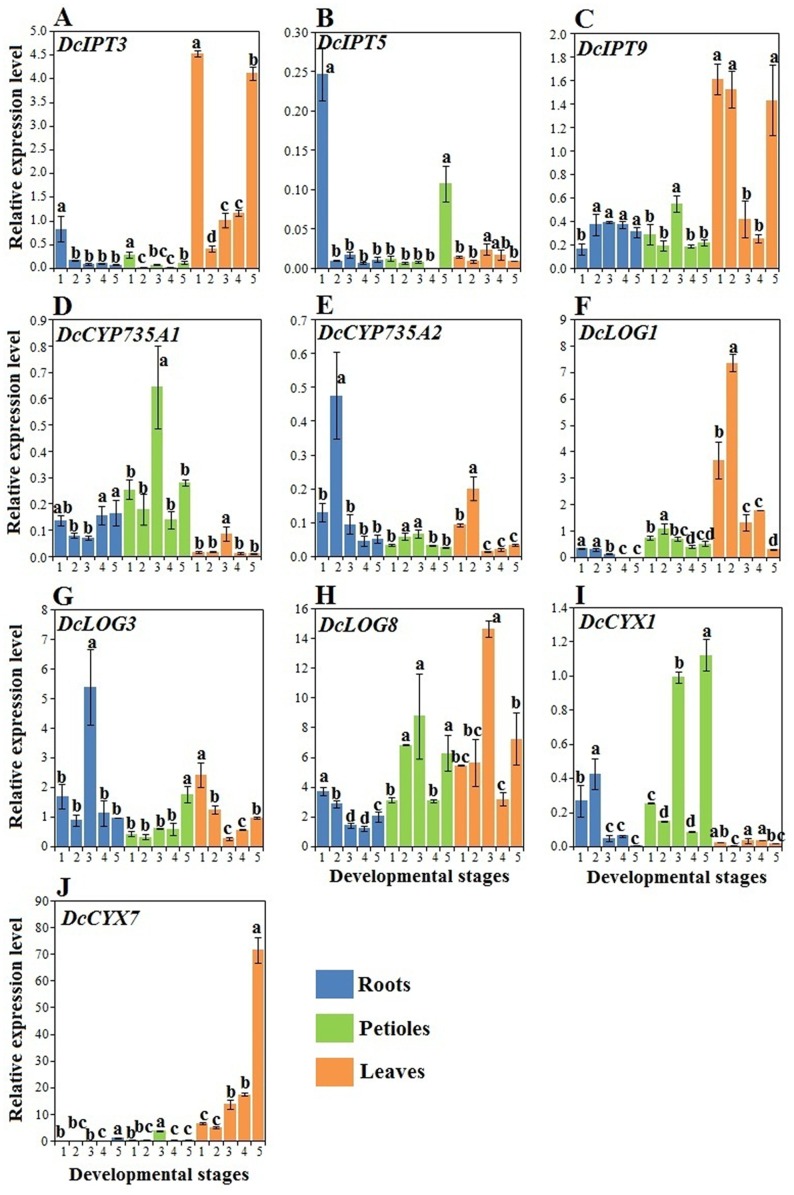
qRT-PCR analysis of genes involved in CK biosynthesis and inactivation among different tissues during carrot growth and development. Error bars represent standard deviation among three independent replicates. Data indicate mean ± SD of three replicates. Different lowercase letters indicate significant differences at *P* <0.05.

*DcIPT3*, *DcIPT5*, *DcIPT9*, *DcCYP735A1*, *DcCYP735A2*, *DcLOG1*, *DcLOG3*, *DcLOG8*, *DcCYX1*, and *DcCYX7* were involved in CK biosynthesis and degradation, as revealed by CarrotDB (Table 1). The transcript levels of these selected genes were evidently altered in response to carrot growth and development (Fig 8).

In the roots, two gene copies of *IPT*, namely, *DcIPT3* and *DcIPT5*, showed higher expression at the first stage (stage 1) and relatively low expression at other stages. By contrast, an opposite trend was detected in the *DcIPT9* gene that encodes tRNA-IPT. *DcCYP735A1* showed the lowest expression at stages 2 and 3, whereas *DcCYP735A2* that encodes CK hydroxylase was highly expressed at stage 2. Transcript levels of *DcLOG1* and *DcLOG8* were higher at the first stage, whereas *DcLOG3* displayed high expression at the third stage and consistently low expression at other stages. In the petioles, the expression profiles of *DcIPT3*, *DcIPT5*, and *DcIPT9* differed from one another during plant growth. The mRNA levels of *DcCYP735A1* and *DcCYP735A2* were the highest at the third stage. A large amount of *DcLOG1* transcript was detected at stage 2, whereas *DcLOG3* was higher at the last stage. *DcCYX1* showed high expression at the last stage, but low expression at stages 2 and 4. In the leaves, *DcIPT9* showed high expression at stages 1, 2 and 5, and low expression at other stages. Conversely, *DcIPT5* was highly expressed at stages 3 and 4. Expression of *DcIPT3* and *DcCYP735A1* in the leaves was similar to that in the petioles. High transcript levels of *DcCYP735A2* and *DcLOG1* were detected at stage 2, whereas *DcCYX7* was highly expressed at the last stage.

Interestingly, some analyzed genes were expressed in a tissue-specific manner. For instance, mRNA abundances of *DcIPT3* and *DcCYX7* were higher in the leaves than in the roots and petioles, whereas an opposite trend was detected in *DcCYX1* expression (Fig 8).

### Expression of CK-responsive genes during carrot growth and development

**Fig 9:**
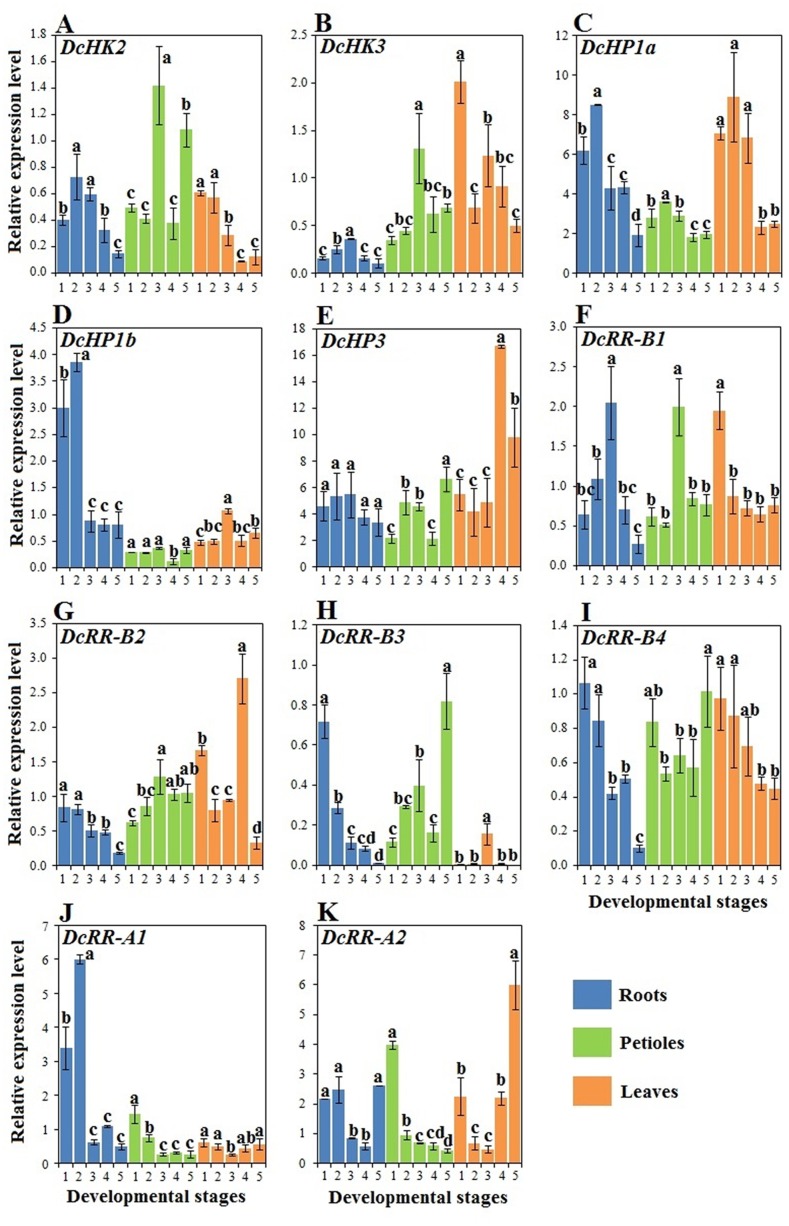
qRT-PCR analysis of genes involved in CK perception among different tissues during carrot growth and development. Error bars represent standard deviation among three independent replicates. Data indicate mean ± SD of three replicates. Different lowercase letters indicate significant differences at *P* <0.05.

CK signal perception and transduction are essential components in the CK network. Thus, the dynamics of CK receptors should be identified to fully understand CK roles during carrot growth. The expression of genes involved in CK response, namely, *DcHK2*, *DcHK3*, *DcHP1a*, *DcHP1b*, *DcHP3*, *DcRR-B1*, *DcRR-B2*, *DcRR-B3*, *DcRR-B4*, *DcRR-A1*, and *DcRR-A2*, were all different from one another (Fig 9).

In the roots, mRNA abundance of *DcHP1b* and *DcRR-A1* showed a pattern similar to that of iP accumulation in the roots. *DcHP3* expression remained relatively stable during plant growth. *DcRR-B2* and *DcRR-B4* exhibited high expression at stages 1 and 2, and low expression at the last stage. *DcHK2* and *DcHP1a* were highly expressed at stage 2, whereas high transcript levels of *DcHK3* and *DcRR-B1* were detected at the third stage. Transcription of *DcRR-B3* decreased over the process of root development. The expression of *DcRR-A2* at stages 1, 2 and 5 was higher than at other stages. In the petioles, high transcript levels of *DcHK2*, *DcHK3*, and *DcRR-B1* were detected at the third stage. Expression of *DcHP1b* showed a pattern opposite to those of iP and ZT. Transcription of *DcRR-B2* increased over the first three stages, and slightly changed at the last two stages. By contrast, the mRNA level of *DcRR-A1* was higher at the first two stages and relatively low at the last three stages. *DcRR-A2* was the only gene that exhibited decreased expression over the developmental stages. In the leaves, mRNA abundance of *DcHK2* was higher at the first two stages. Transcription of *DcHP3* remained relatively low at the first three stages. Conversely, *DcHP1a* and *DcRR-B4* were highly expressed at the first three stages. *DcRR-B1* showed high expression at stage 1 and consistently low expression at other stages. *DcRR-B3* was highly expressed at the third stage, whereas expression of *DcRR-A1* was lowest at this stage. *DcRR-A2* was the only gene that was highly expressed at the last stage.

## Discussion

Plant growth and development is under tight control of both environmental signals and intrinsic cues [37]. Hormones are a class of molecules that can serve as important signals and lead to the induction or repression of the target genes, thereby regulating plant growth [1,38,39]. Among them, CKs can promote cell division in plant roots and shoots, which is mainly achieved by stimulating the production of proteins needed for mitosis [11,40,41]. Thus, a better understanding of CK accumulation and its potential roles during plant growth is of vital importance.

Carrot is a vegetable crop with high nutritional value. ‘Kurodagosun’ is a carrot variety with high and stable production worldwide and has been extensively used in molecular and genetic studies [27,42]. ‘Kurodagosun’ undergoes evident size change during plant growth, which may require the involvement of hormones. However, only limited information regarding CK has been documented in carrot. The results from the current study would substantially improve the understanding of CK accumulation and potential during carrot growth.

As a whole, CK levels differed from different developmental stages and various tissues, indicating that CKs may regulate plant growth in a stage-dependent and tissue-specific manner [10,43]. CKs are assumed to be synthesized not only in the meristem of the roots but also in the shoots [44]. Once CK has been produced, it is translocated to other regions of the plant where continuous growth occurs [45]. CKs are necessary for cell division and may play important roles in vascular development [46,47]. In the present work, we found that CKs were present in all tissues, which may provide constant support for structure formation and development. Our results also showed that higher levels of iP and ZT occurred at the second stage in the roots. Anatomical structure analysis revealed obvious enlargement at this stage (Fig 4). These results led us to hypothesize that the second stage was crucial to root growth, and CKs may play important roles at this stage.

All analyzed genes were expressed among different tissues, supporting the idea that various tissues can produce CKs [10]. The expression of most genes did not follow a pattern similar to that of iP and ZT accumulation. However, these genes are believed to be essentially required for CK accumulation. For example, transcription of *DcCYP735A2* was higher at stage 2 in the roots, which may largely contribute to higher levels of CKs at this stage. Furthermore, CK biosynthesis is regulated not only by transcriptional mechanisms but also by post-translational events [48,49]. Some tissue-specific genes, such as *DcIPT3*, *DcCYP735A1*, and *DcCYX7*, were also identified. All these results suggested that a complex regulatory network for CK metabolism in carrot.

In comparison with biosynthetic routes, CK signal perception and transduction are equally important [50–52]. In *Arabidopsis*, the transcriptional levels of *ARR-A* and *AHP* can be rapidly induced by changes in CK levels, whereas those of others cannot [53,54]. Similarly, *DcHP1b* and *DcRR-A1* showed a pattern similar to that of CK accumulation in roots, whereas transcription of genes encoding type-B RRs was not well correlated with CK accumulation. However, such genes are still integral to the whole pathway. Collectively, the genes encoding the receptors are important to CK signal transduction and normal plant growth and development [52].

## Conclusions

As a carrot grows, significant alterations occur in its morphological and anatomical structure, CK accumulation, and gene expression. CK plays important roles in carrot plant growth, and it may be particularly crucial to root enlargement at the second stage. CK biosynthesis is a complex regulatory network that needs the involvement of many genes. Genes involved in CK perception and transduction are also indispensable to normal plant growth. The current work has substantially improved the understanding of CK accumulation and its potential roles during carrot development.

## Supporting Information

S1 File Relevant data underlying the findings described in the manuscript. **Figures A-U**. Nucleotide acid and deduced amino sequences of CK-related genes. **Table A**. Raw Cq (quantification cycle) values of genes in different tissues during carrot growth and development.
(DOCX)
