# Supplementary material for: Morphological Characteristics, Anatomical Structure, and Gene Expression: Novel Insights into Cytokinin Accumulation during Carrot Growth and Development
Source: PLoS One. 2015 Jul 28;10(7):e0134166. doi: 10.1371/journal.pone.0134166 (PMC4517795; doi:10.1371/journal.pone.0134166)
Supplement: S1 File — Figures A-U. Nucleotide acid and deduced amino sequences of CK-related genes. Table A. Raw Cq (quantification cycle) values of genes in different tissues during carrot growth and development. (DOCX) [file pone.0134166.s001.docx]

**Morphological Characteristics, Anatomical Structure, and Gene Expression: Novel Insights into Cytokinin Accumulation during Carrot Growth and Development**

Guang-Long Wang^1^, Sheng Sun^2^, Guo-Ming Xing^2^, Xue-Jun Wu^1^, Feng Wang^1^, Ai-Sheng Xiong^1,^ *

*1. State Key Laboratory of Crop Genetics and Germplasm Enhancement, College of Horticulture, Nanjing Agricultural University, Nanjing, 210095, China*

*2. College of Horticulture, Shanxi Agricultural University, Taigu, 030801, China*

*Please address all correspondence to: Xiong A.S. ([xiongaisheng@njau.edu.cn](mailto:Xiongaisheng@njau.edu.cn))

*-------------*

Dr. Xiong Aisheng

Professor

State Key Laboratory of Crop Genetics and Germplasm Enhancement,

College of Horticulture,

Nanjing Agricultural University,

Nanjing, 210095, China

Fax: 86 25 84396790

Email: [xiongaisheng@njau.edu.cn](mailto:Xiongaisheng@njau.edu.cn)

**Fig. A** Nucleotide acid and deduced amino acid sequences of *DcIPT3* from carrot

ATGAGGATGTCAATGCTGACGTGCAAACAAATAGCTCCTTCGCTAAGCATACCTGATGGAAGACAGGTTTTGCCA

1 M R M S M L T C K Q I A P S L S I P D G R Q V L P

TTTCTCCGCAGCTCCAGGCCACCAAAAGAAAAGGTTGTGGTTGTCATGGGCGCAACTGGAACCGGCAAGTCTAAG

26 F L R S S R P P K E K V V V V M G A T G T G K S K

CTCTCGATTGACCTAGCCACTCGTTTCAGTGGCGAGGTAGTAAACTCTGACAAGATGCAAGTCTACGAAGGCCTA

51 L S I D L A T R F S G E V V N S D K M Q V Y E G L

GACATAATCACAAACAAGATCACTGAAGAAGAAGCCTGTGATGTACCACACCATCTCTTAGGTATAATTGATCCT

76 D I I T N K I T E E E A C D V P H H L L G I I D P

AATGTGGACTTTACTTCCACAAATTTCTGCAGCATGGCTTCCCTGGCCATAAGATCTATTGCAGGTCGCAGGAAG

101 N V D F T S T N F C S M A S L A I R S I A G R R K

CTCCCAATCATCGTTGGAGGCTCCAATTCATTCATTGAAGCTTTAGTTGATGATGAAACTCACGAATTCCGATCA

126 L P I I V G G S N S F I E A L V D D E T H E F R S

AGGTACGAATGTTGTTTTCTTTGGGTAGATGTCTCAATGCCAGTTCTCCACAGATTTGTATCTGAGAGGGTTGAT

151 R Y E C C F L W V D V S M P V L H R F V S E R V D

AGGATGGTTGAGAATGGAATGGTAGATGAGGCAAGACAAATGTTCAGTTTAGATGCTGATTATTCAAAAGGAGTA

176 R M V E N G M V D E A R Q M F S L D A D Y S K G V

ACAAAAGCAATAGGTTTGCCCGAATTCGATCAGTATTTTCGAGTTGAACCATATGTTAATTTAGAAACTCGAGCA

201 T K A I G L P E F D Q Y F R V E P Y V N L E T R A

AAACTTTGCCAAGAAGCAATAGATGAGGTGAAGAACAACACATGTAAACTAGCCTGCCGCCAGTTAGAGAAGATT

226 K L C Q E A I D E V K N N T C K L A C R Q L E K I

TATCGACTAAGAAACAACAAAGGATGGAAGGTTCACAGACTTGACGCAACAGATGCATTTCTAAAGAATGGCAAA

251 Y R L R N N K G W K V H R L D A T D A F L K N G K

GAATCTGATAAGGCCTGGAACGAGCTCGTGGCGGCACCTAGCATGGTTATTTTAAGCCGGTTTCTTAACAATTTT

276 E S D K A W N E L V A A P S M V I L S R F L N N F

GGACCTAACATATACATGAACCCAACAACTGTCCGTGGGACAGCAATGGAGACAGCCATGGTAACTGCAACTCAT

301 G P N I Y M N P T T V R G T A M E T A M V T A T H

TAG

326 *

**Fig. B** Nucleotide acid and deduced amino acid sequences of *DcIPT5* from carrot

ATGAGGATATCATTTTCTGCCTGCAAACAAGCACCGCCGCAGCCCCTAGTGAATTTCCGCGGCGGAGTTGTGAGC

1 M R I S F S A C K Q A P P Q P L V N F R G G V V S

ATGGACCCCTTCATGCCATGGCGGCGGAAGGACAAAGTCGTGATTCTAATGGGAGCAACCGGCACAGGAAAATCA

26 M D P F M P W R R K D K V V I L M G A T G T G K S

AGACTTTCAATCGACCTAGCCACTTCTTTCCCAGCTGAGATAATCAATTCGGATAAAATTCAAGTCTACGAAGGC

51 R L S I D L A T S F P A E I I N S D K I Q V Y E G

TTAGATATTGTCACAAACAAGGTTACCGAAGAGGAATGTCGAGGCGTGCCGCATCATTTGCTAGGAATGATCCGT

76 L D I V T N K V T E E E C R G V P H H L L G M I R

TCTGACGCAGATTTCACCGCCAATGATTTCCGACACCACGCCACCCTGGCCGTGGAATCCATCGTGAGCCGGGAC

101 S D A D F T A N D F R H H A T L A V E S I V S R D

CGCCTCCCCATCATAGCCGGTGGCTCCAATTCATTTATTAAAGCTCTTGTGAACGACGAATTTAAATCGAGGTAC

126 R L P I I A G G S N S F I K A L V N D E F K S R Y

GCTTGCTGTTTCCTATGGGTTGACGTGTCATTCCCGGTTCTACACTCGTACGTGTCTCATCGAGTCGATAAAATG

151 A C C F L W V D V S F P V L H S Y V S H R V D K M

GTCGAATCCGGGTTAATAGACGAGGTTCGCCAATTTTTCAACCCGGATTTAAATGATTATTCGCGAGGAGTGAGG

176 V E S G L I D E V R Q F F N P D L N D Y S R G V R

CGCGCGATCGGATTGCCAGAAATGGACGAGTTCCTGAGAGCCGAAGCTTGTGTAGATGATGAAACTAGAGATGCT

201 R A I G L P E M D E F L R A E A C V D D E T R D A

CTGCTTGAAATGGCGATCGATAGAATAAAAGGTAACACGTCTTTATTGGCGCTGAAGCAATTAAGAAATATTCGA

226 L L E M A I D R I K G N T S L L A L K Q L R N I R

AGGCTGCGAAAACAAATGGAGTGGGACATGCATCGTTTGGATGCGACCGAGGCTTTTTTAAAGAGTGGATCGGAG

251 R L R K Q M E W D M H R L D A T E A F L K S G S E

TCGCACGAGGCGTGGGAAAGGCGAGTGGGAAGGCCGAGCAGAGGAATTGTTGGTAATTTTCTTTGCGAAGAAGAT

276 S H E A W E R R V G R P S R G I V G N F L C E E D

TCGGCACTATCGGCTTCATTTATTAAAATGAAGGCGTCGTCGCTTGTCGGAGCCGCGGCGGTTAGCACCGCAGTG

301 S A L S A S F I K M K A S S L V G A A A V S T A V

GCTGCCGGCGCCGGTCGGTGA

326 A A G A G R *

**Fig. C** Nucleotide acid and deduced amino acid sequences of *DcIPT9* from carrot

ATGATGAGCGGCGTCGCCGGTGGCTTACGTTTCCGTTGCTTCCATCCCGTCCACCACCTTCGCCGCCGTCAACTC

1 M M S G V A G G L R F R C F H P V H H L R R R Q L

CGCGGCGTCTCCGCCGCCGCGAAAAACAAGGAGAAACCGAAAGTGATAGTCATCTCCGGTCCCACCGGCGCCGGG

26 R G V S A A A K N K E K P K V I V I S G P T G A G

AAAAGTAGACTCGCACTTGAACTCGCTAAGCGACTCAACGGCGAAATCGTTAGCGCCGACTCAGTGCAGGTTTAC

51 K S R L A L E L A K R L N G E I V S A D S V Q V Y

AGAGGCCTTGATGTTGGTTCTGCCAAGCCTTCGTTGTCCGATAGAGAGGAAGTCAAACACCATTTGATTGACATT

76 R G L D V G S A K P S L S D R E E V K H H L I D I

ATGGACCCGTCTGAAGATTACTCTGTCGGGCAATTTTATGAGGAAGCAAGGAATGCTACCAAAGAGATACTTGAA

101 M D P S E D Y S V G Q F Y E E A R N A T K E I L E

AGTGGAAGGGTCCCAATAGTCACTGGTGGAACCGGATTGTACTTAAGATGGTACGTATATGGAAAACCAGATGTC

126 S G R V P I V T G G T G L Y L R W Y V Y G K P D V

CCTAAATCTTCTCCCGAGACTGTGGCTGAAGTACAAGCTGAAATATCAGATTTAGAAAGAGTTGGTGACTGGGAT

151 P K S S P E T V A E V Q A E I S D L E R V G D W D

GCTGCTGTGCAGTTGGTTGTTCAAGCAGGTGATTCAAGTGCTCAATCTTTACCTGCCAATGATTGGTATCGTATA

176 A A V Q L V V Q A G D S S A Q S L P A N D W Y R I

CGTCGCAAGCTTGAGATCATCAAGTCTAGTGGATCGCCTCCTTCAGCTTTTCATGTTCCCTATGATTCATTTAGG

201 R R K L E I I K S S G S P P S A F H V P Y D S F R

GAACAACCTAATTCACCCAATGTAGATGATTTCCGCAGCAGCGCCTCTTTAAAAAATGAATCCCAGGGTAGTAGA

226 E Q P N S P N V D D F R S S A S L K N E S Q G S R

TCTCCAGAGGATTTGGATTACGACTTCTCTTGCTTTTTCTTGTCAAGCCCAAGAATGGACCTCTACAGATCAATT

251 S P E D L D Y D F S C F F L S S P R M D L Y R S I

GACTTTCGGTGTGAAGATATGGTTTCAGGAAGTGACGGGATATTGTCGGAGGCAAAGCGGCTTCTTGATCTTGGT

276 D F R C E D M V S G S D G I L S E A K R L L D L G

CTTCAGCCAAATTCTAATTCTGCAACTCGAGCAATTGGTTACAGAAATGCAATGGAATATCTTTTGCATTGTAAA

301 L Q P N S N S A T R A I G Y R N A M E Y L L H C K

GAACAAGGTGGGAGTTCGACAAGAGACTTTTATGCTTTCTTATCTGGATTCCAGAAAGCATCCAGGAATTTTGCC

326 E Q G G S S T R D F Y A F L S G F Q K A S R N F A

AAGCGACAGTTGACATGGTTCCGTAATGAGCCTATATATAACTGGATCAATGCTTCCAGACCTTTGGAAGATGTG

351 K R Q L T W F R N E P I Y N W I N A S R P L E D V

CTTGGGTTCATCCATGATTCATACCATGTTCGAACTGGAACTATTGAAGTACCTAAAGCACTTCAGATGAAGAAA

376 L G F I H D S Y H V R T G T I E V P K A L Q M K K

GATTTGTCAGAGCGTAGAGAAATCTTACAAATGAAGGGTTATCGTACAAATAATAGGCATTTTGTGGGGCGTGAA

401 D L S E R R E I L Q M K G Y R T N N R H F V G R E

AATTGCGCCGATATTCTAGAATGGATAAGGGAAAGTCAGGGACAGTTAAATTTATCTGTTAACTAG

426 N C A D I L E W I R E S Q G Q L N L S V N *

**Fig. D** Nucleotide acid and deduced amino acid sequences of *DcCYP735A1* from carrot

ATGATGAGCATGTTGATACTAGTAGCATTGTTGGCAGTGGTTCTTCATTTGTTGGTAAGAATCGGCTATGCGAGT

1 M M S M L I L V A L L A V V L H L L V R I G Y A S

CTATCATTTTACTGGCTGACTCCGAGACGCATCAAGAAGATGATGGAAAAGCAAGGAGTGCGTGGCCCTAAAGCC

26 L S F Y W L T P R R I K K M M E K Q G V R G P K A

CGCTTTCTTGTTGGTAACATTTTAGACATGGCTTCTTTCTGTTCTCAATCCACCTCCAAAGACATGGACTCCATT

51 R F L V G N I L D M A S F C S Q S T S K D M D S I

AGTCACGACATCGTTGGTCGCCTTTTGCCGCATTTTGTTGCCTGGTCTAGACTCTACGGGAAGAGATTTATATAT

76 S H D I V G R L L P H F V A W S R L Y G K R F I Y

TGGAATGGGACGGAGCCGAGGATGTGCTTGTCGGAGACGCACCTGATAAAAGAGTTGCTACTAAAACACAGTAGC

101 W N G T E P R M C L S E T H L I K E L L L K H S S

ATATCCGGCAAGTCATGGTTGCAGCAACAAGGCACAAAACATTTCATCGGACGAGGTTTGTTGATGGCTAACGGC

126 I S G K S W L Q Q Q G T K H F I G R G L L M A N G

AACGATTGGTATCATCAGCGTCACATTGTTGCCCCTGCATTCATGGGCGATAAACTCAAGAGTTATGCGGGTTAC

151 N D W Y H Q R H I V A P A F M G D K L K S Y A G Y

ATGGTGGAAAGCACCAAGCAAATGCTGCAATCATTAGAAAATGAAATAAAGTTGGGACAAGCTGAGTTTGAAATT

176 M V E S T K Q M L Q S L E N E I K L G Q A E F E I

GGTGAGTACATGACTCGGCTCACGGCCGACATCATATCTCGAACAGAATTCGATAGTAGCTACGAAAAGGGAAAG

201 G E Y M T R L T A D I I S R T E F D S S Y E K G K

CAAATATTTCATCTGCTCACCGTTTTACAGAATCTGTGTGCACAAGCCAGCCGGCACTTGTGCTTTCCTGGTGGC

226 Q I F H L L T V L Q N L C A Q A S R H L C F P G G

AGGTTTCTACCTAGTAAATACAATAGGGAGATAAAGTCATTGAAAATGGAGGTGGAGAAATTACTGATGGAGATT

251 R F L P S K Y N R E I K S L K M E V E K L L M E I

ATCCAGAGCAGGAAAGACTGCGTGGAGGTGGGGCGAAGCAGTTCATACGGGAATGATTTGTTAGGAATGTTGCTG

276 I Q S R K D C V E V G R S S S Y G N D L L G M L L

GACGAGATGCAGAATAAAACAAGAGGAGGAACTGAGGAATTCAGCCTCAATTTGCAGTTGATCATGGATGAATGC

301 D E M Q N K T R G G T E E F S L N L Q L I M D E C

AAGACTTTCTTTTTTGCAGGACATGATACGACGGCTCTTCTTCTTACATGGACTGTTATGCTCCTTGCTAGCAAT

326 K T F F F A G H D T T A L L L T W T V M L L A S N

CCTTCTTGGCAAGATAAAGTCAGGGCCGAGATTAAGGAAGTCTGCAACGGAAGCTCACTCTCCATTGAACATCTC

351 P S W Q D K V R A E I K E V C N G S S L S I E H L

CCCAAACTTACTCTGTTAAACATGGTGATCAATGAATCGCTTCGCCTTTACCCTCCAGCTTCTGTGCTTCCGAGG

376 P K L T L L N M V I N E S L R L Y P P A S V L P R

ATGGCCTTCGAGGATTATAAGCTCGGAGACCTCCACATTCCAAAAGGATTGTCCATATGGATTCCGGTATTAGCC

401 M A F E D Y K L G D L H I P K G L S I W I P V L A

ATACATCACAGTGAGGAGATATGGGGAAAAGATGTGAATGAGTTCAATCCAGAGAGGTTTGCTTCAAAGACTTTT

426 I H H S E E I W G K D V N E F N P E R F A S K T F

GCTCCTGGCAGACAATACTTCATGCCATTTGCTGCGGGTCCAAGAAACTGCGTTGGTCAATCCTTTGCTTTGATG

451 A P G R Q Y F M P F A A G P R N C V G Q S F A L M

GAAGCTAAGATCATTTTGGGCATGTTAATATCCAAGTTTAGTTTCAACATTTCTCAGAATTACAGGCATGCACCA

476 E A K I I L G M L I S K F S F N I S Q N Y R H A P

GTTATTGTGCTCACCTTGAAACCCAAGTATGGGGTTCAGATATGTTTGAAGCCCTTGGATTCGTGA

501 V I V L T L K P K Y G V Q I C L K P L D S *

**Fig. E** Nucleotide acid and deduced amino acid sequences of *DcCYP735A2* from carrot

ATGGAATTGAACTGGGTTTTCAAAAATGTTGGATTAGCCATCGCGACGATGCTTCTCCTGGTCTTGTGTAAAATA

1 M E L N W V F K N V G L A I A T M L L L V L C K I

GTTTTAAGCTTCTGGTTATGGCCCAACATAGCATACCAGAAGCTTAAAAGGAGTGGCATAAACGGCCCTTCTCCA

26 V L S F W L W P N I A Y Q K L K R S G I N G P S P

AGTTTTCCTATGGGAAACATCACTCATATGGTAGCTATATCAAAGAAAAGCAAACAATCTCCCGTTAATACCAAT

51 S F P M G N I T H M V A I S K K S K Q S P V N T N

TTGACCACCCACGATAATTATTCAACGGTCTTTCCCTACTTTGCTTTATGGCAGAAATCTTTCGGAAAGGTGTTT

76 L T T H D N Y S T V F P Y F A L W Q K S F G K V F

GTGTACTGGCTAGGAACCGAACCATTTTTGTACGTTTCGGATGCGGAGTTTCTGAAGCAAATGAATGCAGCTGTT

101 V Y W L G T E P F L Y V S D A E F L K Q M N A A V

CCGGGGAAGAATTGGGGAAAATCAAATCTTTTCAGAAATGATCGGAAACCAATGTTTGGAAGTGGCTTAGTAATG

126 P G K N W G K S N L F R N D R K P M F G S G L V M

GCTGAAGGTGAGGATTGGGTTCGTCATCGGAATGTTCTTACGCCAGCTTTCTTACCGGCCAACCTTAAGGCCTTG

151 A E G E D W V R H R N V L T P A F L P A N L K A L

GCAAGCTTAATGGTAGCATCAACAAATAATATGATAGACCGATGCACCAACATTATCAACTCTGGGCAGCAAGAA

176 A S L M V A S T N N M I D R C T N I I N S G Q Q E

ATTGATTTCGAAAAAGAAATGATAACGACAACAGGGGAGATTATAGCCAAGACCAGCTTCGGTATGAGCTACGAG

201 I D F E K E M I T T T G E I I A K T S F G M S Y E

AATGGTAGAAAGGTGCTGGAGAGACTAAGAGCTATGCAACAAGCTCTGTTCAATTCCAACCGTTATGTTGGAGTA

226 N G R K V L E R L R A M Q Q A L F N S N R Y V G V

CCATTTAGTAAGTTTTTGTGCCTGGAGAAATACCGGGAAGCAAAAAGACTAGGTGACGAGATTGATGCTCTTCTT

251 P F S K F L C L E K Y R E A K R L G D E I D A L L

CTGGCGCTTATAGAAGACAAAACTAAATCAAAGAAAGATGGAGATCAATCTTGTTTTTCTGCTGATCGTGAGAAG

276 L A L I E D K T K S K K D G D Q S C F S A D R E K

AATTTGTTGGACATTATGCTAGCTGATTACGAGAGTGCTAAGTCGTTAACAACTAAAGAAATGGTGGATGAGTGC

301 N L L D I M L A D Y E S A K S L T T K E M V D E C

AAAACATTTTTCTTCGGTGGCCATGAAACAACAGCTTTGGCACTGACATGGACTCTGTTCCTCTTGGCCGTGCAT

326 K T F F F G G H E T T A L A L T W T L F L L A V H

CCCGAGTGGCAAAATCAACTCAGGGAAGAGATCAAACAAGTTGTAGGAGATCAAGTTGTAGATGCCAGCATGGTT

351 P E W Q N Q L R E E I K Q V V G D Q V V D A S M V

AATAACCTTAAGAAGATGGGGTGGGTAATGAATGAAGCTCTACGATTATATCCGCCTGCGCCTAACCTACAAAGA

376 N N L K K M G W V M N E A L R L Y P P A P N L Q R

CAAGCAAGAGATAACATTCAGGTAAACGAGGTGATTATCCCTAAAGACACCAATATCTTGATTGATGTTATGGCA

401 Q A R D N I Q V N E V I I P K D T N I L I D V M A

ATTATGCACGATCGTGGCTTTTGGGGCGACACAGTCCACCAATTTAGGCCTGAGAGATTTGAGCCTGATAACTTA

426 I M H D R G F W G D T V H Q F R P E R F E P D N L

TATGGAGGATGCGAACACAAGATGGGGTATGTGCCTTTTGGTTTTGGAGGAAGAATGTGCATTGGTAGAAACCTT

451 Y G G C E H K M G Y V P F G F G G R M C I G R N L

GCAATCATGGAGTACAAGATTGTGTTAACCTTAATCTTGAGTAGGTTTTCGTTCTCGCTTTCACCTTTCTACACT

476 A I M E Y K I V L T L I L S R F S F S L S P F Y T

CATTCCCCTGCCATCATGTTGTCTCTTAGGCCTGCCAAGGGAATGCCCCTCGTGGTTCAACCTTTGTATTAG

501 H S P A I M L S L R P A K G M P L V V Q P L Y *

**Fig. F** Nucleotide acid and deduced amino acid sequences of *DcLOG1* from carrot

ATGGAAGGGGATGAAGCAGAGACATCGACGAGTCATGATAAGAAGATAGTTTTACGAAAGTTCAGAAGTATATGT

1 M E G D E A E T S T S H D K K I V L R K F R S I C

GTGTTTTGTGGGAGTAGAGCTGGTTATAATTCTTCATTCACTCATGCTGCTCTGCAGCTTGGTAAACTCATGGTT

26 V F C G S R A G Y N S S F T H A A L Q L G K L M V

GAGCGGAAGATTAATTTGGTTTATGGTGGAGGAAGCATTGGCTTGATGGGAATGATTGCTGAAACTGTTTACAAA

51 E R K I N L V Y G G G S I G L M G M I A E T V Y K

GGTGGTTGCCATGTTCTTGGAGTGATACCTAAAGCTTTAGTGGCAGATGAGATTTCAGGAAAAACAATCGGAGAT

76 G G C H V L G V I P K A L V A D E I S G K T I G D

GTGAAGATAGTGGGAGATATGCATCAAAGAAAGTCTGAGATGTCTAAGCATGCTGATGCCTTCATAGCACTTCCT

101 V K I V G D M H Q R K S E M S K H A D A F I A L P

GGAGGATATGGAACCATGGAAGAATTGCTAGAGGTGATCACATGGTCTCAGCTAGGAATCCATGAAAAACCAGTG

126 G G Y G T M E E L L E V I T W S Q L G I H E K P V

GGGTTGCTAAATGTGGATGGCTATTATGATAGCCTGCTTGCCTTGTTTGACAAAGGAGTGGAAGATGGTTTCATA

151 G L L N V D G Y Y D S L L A L F D K G V E D G F I

GATGATTCAGCCAGAAATATTGTGGTCTCAGCAAATACACCACAAGACCTGATCAGCAAAATGGAGAATTATGTG

176 D D S A R N I V V S A N T P Q D L I S K M E N Y V

GCAGTCCATGAGATAGTAGCTTCAAGACAAAGTTGGGAAGTGGACCAATCATTAGAGGCTACCACAAGTGGGGAA

201 A V H E I V A S R Q S W E V D Q S L E A T T S G E

CTTGTTTAA

226 L V *

**Fig. G** Nucleotide acid and deduced amino acid sequences of *DcLOG3* from carrot

ATGGAGAGACAGGGTGAGATGCAGCAATCAAAATTTGGAAGGATTTGTGTTTTCTGTGGGAGTAGTCAAGGCAAA

1 M E R Q G E M Q Q S K F G R I C V F C G S S Q G K

AAGACTAGTTACCAAGATGCTGCCATTCAGCTTGGCCAAGAATTGGTGTCAAGAAATATTGATTTGGTGTATGGA

26 K T S Y Q D A A I Q L G Q E L V S R N I D L V Y G

GGGGGGAGCATTGGCCTAATGGGATTGATTTCACAAGCTGTACACAATGGTGGTCGTCATGTCATTGGGGTCATT

51 G G S I G L M G L I S Q A V H N G G R H V I G V I

CCCAAGACACTCATGCCTCGAGAGTTAACTGGTGTAACAGTAGGGGAGGTGAAGGCAGTTGCAGGCATGCATCAA

76 P K T L M P R E L T G V T V G E V K A V A G M H Q

AGAAAAGCAGAGATGGCTAGGCACTCAGATGCTTTTATTGCCTTGCCAGGTGGTTATGGTACTCTTGAGGAGCTC

101 R K A E M A R H S D A F I A L P G G Y G T L E E L

CTTGAAGTTATAACTTGGGCACAACTCGGTATCCATGATAAACCGGTGGGGTTGCTCAATGTGGACGGATACTAC

126 L E V I T W A Q L G I H D K P V G L L N V D G Y Y

AACTCTTTACTGTCATTTATTGACAAAGCCGTGGAAGAAGGCTTCATCAGCCCTAATGCACGCCATATTATTATA

151 N S L L S F I D K A V E E G F I S P N A R H I I I

TCTGCACCAACAGCAAAGGATTTGGTCAAGAAACTGGAGGAATATGTACCATGCCATGAAAGGGTTGCTTCAAAG

176 S A P T A K D L V K K L E E Y V P C H E R V A S K

CTGAATTGGGAGACAGAGCGGCTTGCCTTCCCGCAAGCGTATAATACCTTAAGATGA

201 L N W E T E R L A F P Q A Y N T L R *

**Fig. H** Nucleotide acid and deduced amino acid sequences of *DcLOG8* from carrot

ATGGAAGGCAATACAAGTGGGAGATTCAAGAGAATCTGTGTCTTCTGTGGAAGCCATCCTGGCCGTAGAAAGGTT

1 M E G N T S G R F K R I C V F C G S H P G R R K V

TTCAGTGATGCTGCTCTTGACTTGGGAGATGAACTGGTTGATAGAAAGATTGACTTGGTGTATGGTGGTGGTAGT

26 F S D A A L D L G D E L V D R K I D L V Y G G G S

GTTGGTTTGATGGGAACCATTTCCCAGAGAGTTTACGAGGGAAAATGTCATGTACTTGGGATTATTCCTAAAGCT

51 V G L M G T I S Q R V Y E G K C H V L G I I P K A

CTTGTGCCTCTGGAGATATCTGGCGAGACTGTTGGAGATGTAAGAATTGTTTCAGACATGCATGAGCGTAAAGCT

76 L V P L E I S G E T V G D V R I V S D M H E R K A

GAAATGGCGCGAGAAGCTGATGCTTTCATTGCTCTTCCAGGAGGATATGGAACCATGGAAGAACTATTGGAGATG

101 E M A R E A D A F I A L P G G Y G T M E E L L E M

ATCACTTGGTCTCAACTTGGAATTCATAAAAAGCCGATTGGTCTACTAAATGTTGATGGTTATTACAACTCTTTG

126 I T W S Q L G I H K K P I G L L N V D G Y Y N S L

CTAGCGTTATTTGACACCGGTGTAGAAGAAGGTTTCATCAAGCCCAGTGCTCGGGACATAATTCTCTCAGCTCCA

151 L A L F D T G V E E G F I K P S A R D I I L S A P

AGTGCCAAAGAACTTTTGATACAGATGGAGCAATATATTCCTTCCCATGACAATGTTGCTCCTCATGAGAGCTGG

176 S A K E L L I Q M E Q Y I P S H D N V A P H E S W

GGGATGGAGGAGCTGGGCAAGTATCCAAAGTAA

201 G M E E L G K Y P K *

**Fig. I** Nucleotide acid and deduced amino acid sequences of *DcCYX1* from carrot

ATGAAATATTTATGTGTAAAAGTCTTTATGGTCTTGTTCTTAACCTCTCTAGCAATTAAGCTGCACTTTTGTTTT

1 M K Y L C V K V F M V L F L T S L A I K L H F C F

CCTAGTATTCCACTATCACTCAGGACAGTTGTTCTGGATGGACACCTCAGTTTCACAGATAATGAATTCGCAGCC

26 P S I P L S L R T V V L D G H L S F T D N E F A A

AAAGACTTTGGTGATCAGTACCGTTACTCTCCGCTAGCAGTCCTTCATCCAAAGTCAGTTTCTGATATTTCTACT

51 K D F G D Q Y R Y S P L A V L H P K S V S D I S T

ATCATAAAGCATGTTTGGCAGATGGGTCCATATACGGAGCTCAAAATTGCAGCTAGAGGCCGCGGTCACTCACTT

76 I I K H V W Q M G P Y T E L K I A A R G R G H S L

CACGGTCAGTCACAAGCTAACCGAGGAATTGTTATTAGTATGGAGTCACTCTGGAGACAAAAAATGCAGTTTCAC

101 H G Q S Q A N R G I V I S M E S L W R Q K M Q F H

ATCGGAAAAACTTGTTATGTGGATGTTTCTGGTGGTGCACTGTGGATAAATATACTGCATGAAAGCCTAAAATAT

126 I G K T C Y V D V S G G A L W I N I L H E S L K Y

GGCTTAACACCAAAGTCTTGGACAGATTATTTACATCTCAGTGTTGGTGGTACTTTGTCCAATGCAGGAATCAGT

151 G L T P K S W T D Y L H L S V G G T L S N A G I S

GGTCAGGCTTTTCGACATGGTCCACAGATCAATAATGTACATCAGCTGGAAGTTGTCACAGGAAAAGGGGAAGTG

176 G Q A F R H G P Q I N N V H Q L E V V T G K G E V

GTAAATTGTTCAGAAAGTCAGAACACAGACCTCTTTTATGGTGTTCTCGGAGGACTTGGTCAGTTTGGTGTAATA

201 V N C S E S Q N T D L F Y G V L G G L G Q F G V I

ACCCGCGCAAGAATATCATTACAACCTGCACCGCATAAGGTCAAATGGATCAGAGTGCTGTACTCAGACTTCTCA

226 T R A R I S L Q P A P H K V K W I R V L Y S D F S

ACATTCTCCAAAGACCAAGAATATCTGATTTCTGTTGAAAAAACATTTGATTACATTGAAGGACTTGTAATAAAG

251 T F S K D Q E Y L I S V E K T F D Y I E G L V I K

AACAAAACAAATCTTATGAATGATTGGAGATCAAACTTCACCCCACAAGATTCAGTTCGAGCCAGTCAATTCATA

276 N K T N L M N D W R S N F T P Q D S V R A S Q F I

TCAGAGGGGAAACTCCTATTTTGTCTGGAGTTGGCGAAAAACTTCAACCCAGAGGAAACAGAATCAACTAATAAG

301 S E G K L L F C L E L A K N F N P E E T E S T N K

GAAATACAGAGGTTACTGTCTCAACTATCTTATATCTCTTCCACACTTTTTACGACAGAAGTTTCATATGTGGAG

326 E I Q R L L S Q L S Y I S S T L F T T E V S Y V E

TTTCTAGACAGAGTTCATACATCCGAGATCAAACTACAATCAAAAGGCTTGTGGGAAGTTCCACACCCCTGGCTC

351 F L D R V H T S E I K L Q S K G L W E V P H P W L

AATCTCTTCATTCCAAAAAGCAAAATCAACAAGTTTGCTCAAGAAGCCTTTGGCAACTTGCTAAAGGATACAAAT

376 N L F I P K S K I N K F A Q E A F G N L L K D T N

AATGGACCTATTCTTGTCTATCCAGTAAACAAATCAAAGTGGAATAACAGAACTTCTTTGGTTCTTCCAGATGAA

401 N G P I L V Y P V N K S K W N N R T S L V L P D E

GATATAATCTACCTAGTGGCATTCCTATCTCATGCAGTTCCCTTACCAAATGGATCAGACAGCTTAGACCATATA

426 D I I Y L V A F L S H A V P L P N G S D S L D H I

TTAAAACGGAACAAACAAATTCTAGAATTTTGCGAGAGTGCACAACTTGGAGTAAAGCAGTATCTGCCACATTAC

451 L K R N K Q I L E F C E S A Q L G V K Q Y L P H Y

CACAAGCAAGAAGAGTGGATGCACCATTTTGGTCCCAAATGGGAAGTCTTTGCAAAGAGAAAATCAGCTTACGAC

476 H K Q E E W M H H F G P K W E V F A K R K S A Y D

CCTCTGGCACTTCTTGCTCCTGGACAGAGAATATTTCAGAACAATTTATCGTACTTATGA

501 P L A L L A P G Q R I F Q N N L S Y L *

**Fig. J** Nucleotide acid and deduced amino acid sequences of *DcCYX7* from carrot

ATGATAGCTTACTTAGAATATCTCCTCCAAGAAAACCAACCGGAAACAATGGCAGATTCCGATGAAATTCCCCTC

1 M I A Y L E Y L L Q E N Q P E T M A D S D E I P L

CTCTCCGCCATTGATCTCCGCGGCACCATCGAACATAATTCCCCGCTTGCCGCCACCGACTTCGGCGGCATGCAA

26 L S A I D L R G T I E H N S P L A A T D F G G M Q

AACACTAAACCGCTGGCCTTCATTCATCCTGCCGGCTCTGATGATATATCGAAAATCATCAGACACGCCGGCAGG

51 N T K P L A F I H P A G S D D I S K I I R H A G R

TTCAGTAGTATGACCGTTGCCGCGAGGGGCAACGGTCATTCTATTAACGGCCAGGCCATGTCAAATAATGGTCTG

76 F S S M T V A A R G N G H S I N G Q A M S N N G L

GTCGTTAACATGAAAACTATCAAGAAAATTTGCGTTAAGCGCGTAAATTTTCGCGGTAACGCCACCTACGTTGTT

101 V V N M K T I K K I C V K R V N F R G N A T Y V V

GATGTAGGTGGCGGAGCACTGTGGGAGGAGGTGCTGAAACGGTGCGTATTGGAGCATGGACTTGCGCCGAGGTCG

126 D V G G G A L W E E V L K R C V L E H G L A P R S

TGGACGGATTATCTAGGGCTGACGGTTGGCGGGACGTTATCGAACGGCGGCGTGAGTGGACAAGCTTTTCGATAC

151 W T D Y L G L T V G G T L S N G G V S G Q A F R Y

GGTCCTCAAACTTCGAATGTGATCGAAATGGAAGTTGTTACGGGGAATGGTGAGGTAGTGATTTGTTCCGAGAAT

176 G P Q T S N V I E M E V V T G N G E V V I C S E N

CAGAGCTCGGATGTTTTCTATTCGGTTCTCGGAGGTCTTGGACAGTTTGGTGTCATTACTCGAGCTAGGGTTTTG

201 Q S S D V F Y S V L G G L G Q F G V I T R A R V L

CTTCAACCGGCCCCGGAAATGGTGAGATGGATAAGGCTAGTTTATTCGAATTTCGATGAGTATACCAATGATGCC

226 L Q P A P E M V R W I R L V Y S N F D E Y T N D A

GAATTTTTGGTCAGTCGGTCGGAAAGCGATGAATCGTTTGATTATGTGGAGGGATTTGTGTTTACGAATAATGAT

251 E F L V S R S E S D E S F D Y V E G F V F T N N D

GATCCGGTGAATGGCTGGCAATCGGTGCCGCTGAATTCGGACCAGATATTCGATCCGACCCTAGTCCCGTGCTAT

276 D P V N G W Q S V P L N S D Q I F D P T L V P C Y

GCTGGACCTGTTCTGTACTGTCTGGAGGTCGCATTGCATTACAGTAAATCCGACGAGCCCTCAGCCGTTGACATG

301 A G P V L Y C L E V A L H Y S K S D E P S A V D M

GTTGTGGAGAGATTGGTTCGACGGCTGAGTTTTGTGGAGGGTCTGAGATATGAAAGGGAGCTGAGCTACATGGAG

326 V V E R L V R R L S F V E G L R Y E R E L S Y M E

TTTCTTTTGAGAGTAAAACGTGACGAACAACAAGCTAGAGCTAACGGTATTTGGGACGCCCCACATCCTTGGCTC

351 F L L R V K R D E Q Q A R A N G I W D A P H P W L

AACTTGTTCGTGTCGAAAACAGACATTGCAGCATTTAATCATCTCATCTTTAACCAAATCTTGAGTCATGGAATC

376 N L F V S K T D I A A F N H L I F N Q I L S H G I

GGTGGCCCCATGCTCGTGTACCCGCTTCTTCGTTCCAAGTGGGATAATCGAACATCAGTGGTGTTACCAGAAGGG

401 G G P M L V Y P L L R S K W D N R T S V V L P E G

GAGGTATTCTACCTAGTAGCATTACTCAGATTTACTCTGCCATATCCAAAGGGACCTCCAGTGGAAGAATTGATC

426 E V F Y L V A L L R F T L P Y P K G P P V E E L I

TCCCAGAACGACGAAATTATCAACTGTTGCATAAGAAACGGCTTCGATTTCAAGCAATATTTCCCTCACTACAAT

451 S Q N D E I I N C C I R N G F D F K Q Y F P H Y N

TCAGAGGAAGGATGGAAGCAACATTTTGGAAATCAGTGGTCACGATTCGTAGAAAGGAAGGCTAAATACGATCCA

476 S E E G W K Q H F G N Q W S R F V E R K A K Y D P

AAGGCCATTCTTGCACCTGGACAGAAGATTTTCAGGAGAAATCGTCGTGAATCCTAG

501 K A I L A P G Q K I F R R N R R E S *

**Fig. K** Nucleotide acid and deduced amino acid sequences of *DcHK2* from carrot

ATGAGTTCTCTTGATCTGTTTGGAGTTCCCCTTAAGCTTTCCAGGATCTTTTTGAAGATATGTAAGTGGATTTTG

1 M S S L D L F G V P L K L S R I F L K I C K W I L

TTAAAAATGTCTTTGAATTCTAAGGTCGGTTCAAATAGCAAGTTGCCTGCTAATTTTAAGCTGAAAAAGGCAAAT

26 L K M S L N S K V G S N S K L P A N F K L K K A N

GAACTCCAGAATGGATTAGGTTATGGCTATCTACGGAGGGCTCTACTTTCCTCTGTATTTCTTGTTGTTCTAGTT

51 E L Q N G L G Y G Y L R R A L L S S V F L V V L V

GGGCTAGTTTGGTTCTTGTTCTGCACGGAAAAGGGGGAATTGCAGACGAAGCTGGAGACACCAGTTTTTTGTGAT

76 G L V W F L F C T E K G E L Q T K L E T P V F C D

AGCAATTCTGGGGTCTTGCATGAACATTTTAATGTTAGCAAAGAAGAATTTGATGTCTTGACCTCTTCTTTCTAT

101 S N S G V L H E H F N V S K E E F D V L T S S F Y

GAATTGGATCAGATGGCATGCTTAAAATGTACCAAACAGTCCAATACGATTCACCATCCTAGTAGTGGTATCAAT

126 E L D Q M A C L K C T K Q S N T I H H P S S G I N

TGTGACTTTGTTATGGCAACGTTAGATCACCAAAGATTTAAGAAAGAAGAGTTGGAAATGACGAATGTAGGATTG

151 C D F V M A T L D H Q R F K K E E L E M T N V G L

CAAGAACAATGCCCTGTCCCAGCTGAGAACACTGACTCATTATTGAAGGAGGGCAAGTCTGTATCAGAGATTTTG

176 Q E Q C P V P A E N T D S L L K E G K S V S E I L

CATTCCATATTTTCATCTATCAAGTCAAAGTTTTGGAACGATCAAGAGTATATGCATTTGAGGGAACCAGAGAAT

201 H S I F S S I K S K F W N D Q E Y M H L R E P E N

TTAGTGACAGAGCATTGCAAGGGTATTTCTTTTTGTTTGACGAATATACTCCTGTGCGCTCTTGTTGGATCAGCA

226 L V T E H C K G I S F C L T N I L L C A L V G S A

ATCTGCTGTCCGATTTTTGGTTTTATTTTTAAATTCTGGAGGAGACGGAAGGTGATGCCTCTTCATCAACATTCA

251 I C C P I F G F I F K F W R R R K V M P L H Q H S

TTGCATCAAAACCAGCAACTCCAAGTGGTCCAGCAAAAGCAACAACAACAATCTCCAATTTCTGGAAAGTGGAGG

276 L H Q N Q Q L Q V V Q Q K Q Q Q Q S P I S G K W R

ATGAAGCTACTACTGGCTTTTGTCATTGCAGGGGTCACTGGGTCCATTTGGTTATTCTGGCACCTAAATGAAGAT

301 M K L L L A F V I A G V T G S I W L F W H L N E D

ATTGTTTTGAGAAGACAAGAAACATTGTCAAATATGTGTGACGAGCGAGCACGTATGCTACAGGATCAATTTAAT

326 I V L R R Q E T L S N M C D E R A R M L Q D Q F N

GTGAGTATGAACCATGTTCATGCATTGGCTATTCTAATCTCCACATTTCATCATGGAAAACAACCTTCTGCTATT

351 V S M N H V H A L A I L I S T F H H G K Q P S A I

GACCAGAAAACATTTGGAGAATATACTGAGAGAACTGCTTTTGAAAGGCCTCTTACTAGTGGTGTTGCTTATGCT

376 D Q K T F G E Y T E R T A F E R P L T S G V A Y A

TTGAGAGTTCCACACTCTCAAAGAGAGCAATTTGAAACTCAACATGGGTGGAAGATTAAAAAAATGGAAACTGAG

401 L R V P H S Q R E Q F E T Q H G W K I K K M E T E

GATCAAACATTAGTTCAAGACTGCATTCCAGAGAATTTAGATCCTGCACCAGTTCAAGATGAATATGCACCAGTT

426 D Q T L V Q D C I P E N L D P A P V Q D E Y A P V

ATATTTTCTCAAGAAACAGTCTCGCATATTGTTTCTATTGATATGATGTCTGGCAAGGAAGATCGGGAGAACATT

451 I F S Q E T V S H I V S I D M M S G K E D R E N I

TTACGGGCTAGGGCATCTGGAAAGGGTGTCTTGACATCTCCTTTTAAGCTATTAAAATCCAATCACCTGGGTGTT

476 L R A R A S G K G V L T S P F K L L K S N H L G V

GTACTTACTTTTGCTGTCTACAACACTCATCTACCTCCTCATGCTACTGCTGAGCAATTTATTAATGCTACTGTT

501 V L T F A V Y N T H L P P H A T A E Q F I N A T V

GGGTATTTAGGTGCATCCTATGATGTCCCGTCACTAGTGGAAAAGCTTCTGCATCAACTTGCTAGCAAACAAACC

526 G Y L G A S Y D V P S L V E K L L H Q L A S K Q T

ATTGTTGTGAATGTTTATGACACAACAAATAAAGCAGAGCCAATAAATATGTACGGTACCAATGTTACCGACACA

551 I V V N V Y D T T N K A E P I N M Y G T N V T D T

GGACTGCTGCACATTAGCACACTTGACTTCGGGGATCCAACTAGAAAACATGAAATGCATTGCAGGTTTAAGCAG

576 G L L H I S T L D F G D P T R K H E M H C R F K Q

AGGGCCCCGCCACCTTGGACAGCAATAACAGCATCTGTTGGTGTTCTTGTAATAACTTTGCTTCTGGGTCATATA

601 R A P P P W T A I T A S V G V L V I T L L L G H I

TTCCGTGCAGCCATAACCCGCATTGCTGAAGCTGAGCTAGGCTACCAAAATATGAGAGTACTTATGCATCGTGCT

626 F R A A I T R I A E A E L G Y Q N M R V L M H R A

GAAGCTGCAGATAAAGCAAAATCTCAGTTTCTGGCAACAGTTTCACATGAAATCAGGACTCCAATGAATGGTGTT

651 E A A D K A K S Q F L A T V S H E I R T P M N G V

TTAGGCATGCTTCAGATGCTTATGGACACAAATCTTGATGCCAACCAACTAGATTATGCACAGACTGCCCATGCT

676 L G M L Q M L M D T N L D A N Q L D Y A Q T A H A

AGTGGAAGAGATCTTATATCACTTATTAATGAGGTTTTAGATCAGGCTAAAATTGAATCTGGCAGACTTGAGCTT

701 S G R D L I S L I N E V L D Q A K I E S G R L E L

GAGGCTGTTCCTTTTGATCTTCGGGCTGTTCTTGATAATGTTTTATCACTTTTCTCGACCAAATCTCATGAGAAA

726 E A V P F D L R A V L D N V L S L F S T K S H E K

GGAATTGAGTTGGCCGTTTATGTTTCTAATCAAGTTCCTGAAATCGTCATCGGAGACCCTGGACGGTTGCGGCAA

751 G I E L A V Y V S N Q V P E I V I G D P G R L R Q

ATAATCACAAATCTTGTTGGAAACTCAATTAAGTTTACACAAGACAGAGGACACATTTTTGTATCAGTGCATCTA

776 I I T N L V G N S I K F T Q D R G H I F V S V H L

GCAGATGAAGTTGGGTACCCACTCGAGGAGCAGGATGAAGTGCTGAGACCAAACATTACTCTGGTTGAAAATTGT

801 A D E V G Y P L E E Q D E V L R P N I T L V E N C

ATAAACAATTCTCGTAACACATTAAGTGGGTTTCCTGTGGTTAATCGATGGAAAAGTTGGAAGGGTTTTGGAAAT

826 I N N S R N T L S G F P V V N R W K S W K G F G N

TTGAATGTGGGTGAATTGTCAAAGGAAACAGAAATGGTCAAAGTATTGGTTACTGTTGAAGATACAGGGGTTGGT

851 L N V G E L S K E T E M V K V L V T V E D T G V G

ATACCACAAGAAGCACAAGGTCATATATTCATGCCCTTTATGCAGGCTGACAGTTCTACATCACGAACATATGGT

876 I P Q E A Q G H I F M P F M Q A D S S T S R T Y G

GGAACCGGGATAGGATTGAGTATAAGCAAGAGGCTGGTGGACCTGATGGGTGGAGAAATTGGCTTTGATAGTGAG

901 G T G I G L S I S K R L V D L M G G E I G F D S E

CCTGGAACAGGCAGCACCTTTTCATTCAGTGTAGCTTTCAAAAAAGGAGAACAAAATTCTCCTGATTTGAAGTCT

926 P G T G S T F S F S V A F K K G E Q N S P D L K S

CCACAGTACCGTCCAACTGTTTCAGAATTTCAAGGATTGAGAGCATTGGTGATTGATGGGAAAAGCATTCGAGCT

951 P Q Y R P T V S E F Q G L R A L V I D G K S I R A

GAAGTCACAAGATATCATCTTCAGAGATTGGGAATTTCAGTAGAAAAAGCTTCGACTCTGGATTCTGCGTATGCT

976 E V T R Y H L Q R L G I S V E K A S T L D S A Y A

TCTCTCACTTGCAACTCCAAGACAAGCATATCGGGACAGTTGGCCATGATTCTTGTTGACCAGGATGTATTAGAT

1001 S L T C N S K T S I S G Q L A M I L V D Q D V L D

GAGATGACTGATACTTCTTACAACAAAATTCTTAAAGAACTGAGACCAAATAGAAGTTCAAGCATCCTAGAAATT

1026 E M T D T S Y N K I L K E L R P N R S S S I L E I

GGTCCAAAAATACTTCTGATGGCACCCTCTAGTTCTGAAAAGTGTAACAAACTCAAGTCGGCTGGGTTGGTTGAT

1051 G P K I L L M A P S S S E K C N K L K S A G L V D

GCCGTTCTAACAAAGCCACTTCGGCTAAGTGTGTTAATTCTTTCATTCCAAGAAACCCTTAGTTTTGACAAGAAG

1076 A V L T K P L R L S V L I L S F Q E T L S F D K K

AGGCCCCCTTCAAGAAGTAAACCATCAACTCTTGGAAATCTACTCAGAGACAAGCGTATCTTGGTGGTAGATGAT

1101 R P P S R S K P S T L G N L L R D K R I L V V D D

AACATGGTTAACAGACGAGTTGCTGAAGGTGCCTTGAAGAAGTATGGAGCAGTTGTCACCTGTGTTGATAGTGGG

1126 N M V N R R V A E G A L K K Y G A V V T C V D S G

AGATCTTCATTAAAGATGCTTAATCCCCCCCACAACTTTGATGCTTGCTTTATGGATCTCCAAATGCCAGAAATG

1151 R S S L K M L N P P H N F D A C F M D L Q M P E M

GATGGGTTTGAAGCTACACGGCAGATCCGCTGTTTGGAGAGCGAAGTAAATAAAAAAATTAATTCTGGTGAAGCA

1176 D G F E A T R Q I R C L E S E V N K K I N S G E A

TCGATTGAGATGTACGGTAATGTGGCTCACTGGCATACACCAATATTAGCAATGACAGCTGATGTAATTCAAGCA

1201 S I E M Y G N V A H W H T P I L A M T A D V I Q A

ACAAATGAGGAGTGCACAAAATGCGGGATGGATGGTTATGTGTCAAAGCCATTCGAAGAAGAAGAACTATATTCA

1226 T N E E C T K C G M D G Y V S K P F E E E E L Y S

GCTGCAGCGCGCTTCTTTGATTCAGGTTAA

1251 A A A R F F D S G *

**Fig. L** Nucleotide acid and deduced amino acid sequences of *DcHK3* from carrot

ATGAGTTATTTTTATGTTGTTGGGTTTGCTCTGAAGGTGGGGCAGTTGCTGTTGATGCTATGGGGATGGATAATA

1 M S Y F Y V V G F A L K V G Q L L L M L W G W I I

TCATTGATTTCAATGAGCTGGTTCATTAATGGAGGAATTATGAGCACTAAGAATGATTTGCTTGGGGATGGGAGG

26 S L I S M S W F I N G G I M S T K N D L L G D G R

AAGCTATGGCTGGTGTGGTGGGATAAGATCAGTGCTTATAAGATTTATAATTTCTATTTTGAGTATTTTAGTCCA

51 K L W L V W W D K I S A Y K I Y N F Y F E Y F S P

AAGAGATTTCGAAAAAGCTGGTGGAGGAATCTTTTGGTGACATGGGTGCTGATTGGAACCTTGTTTTCGATGTGG

76 K R F R K S W W R N L L V T W V L I G T L F S M W

GTGTTTTGGTATATGAACTCTCAAGCTTTGGAGAAAAGAAAGGAGACTCTTGCAAGTATGTGTGATGAGAGGGCT

101 V F W Y M N S Q A L E K R K E T L A S M C D E R A

AGAATGTTACAAGATCAGTTTAATGTGAGTATGAATCATGTCCAAGCTATGTCTATCTTGATTTCAACCTTTCAC

126 R M L Q D Q F N V S M N H V Q A M S I L I S T F H

CATGCCAAGAATCCCTCTGCTATCGATCAGGGGACCTTTGCCAGGTACACTGAAAGAACAGCTTTTGAGAGGCCC

151 H A K N P S A I D Q G T F A R Y T E R T A F E R P

CTTACAAGTGGTGTAGCATATGCTGTAAGGGTGCTCCACTCAGAAAGAGAACATTTTGAGAAGCAGCAAGGCTGG

176 L T S G V A Y A V R V L H S E R E H F E K Q Q G W

AAAATTAAGAGGATGGATCATTCTGATCAAGTCCCAGTTCCTAAGGGTGTATATGATGGGGAAGAATCCGAAGCC

201 K I K R M D H S D Q V P V P K G V Y D G E E S E A

CCAGAGCCATCTTTAATTCAGCCAAATCAGGAGGAATATGCTCCAGTTATTTTTGCTCAGGAGACTGTTGCTCAC

226 P E P S L I Q P N Q E E Y A P V I F A Q E T V A H

GTGGTTTCCATTGATATGCTGTCAGGGAAGGAAGATCGTGATAATATCTTGCGTGCAAGAGAATCTGGGAAAGGG

251 V V S I D M L S G K E D R D N I L R A R E S G K G

GTTCTCACTGCTCCTTTCAAGCTGCTCAAATCAAATCGATTAGGTGTCATACTGACTTTTGCCGTCTACAAGATA

276 V L T A P F K L L K S N R L G V I L T F A V Y K I

GATTTTCCTTCTAATGCAACACCAGATGAGAGAGTCCAAGCAACTGATGGGTACCTTGGTGGAATATTTGATATT

301 D F P S N A T P D E R V Q A T D G Y L G G I F D I

GAATCGCTTGTAGAAAAGTTACTTCAACAGCTTGCGAGCAAACAAACAATACTCGTGAATGTCTATGATACCACC

326 E S L V E K L L Q Q L A S K Q T I L V N V Y D T T

AACCTATCTTCCCCTATCAGCATGTACGGTTCAAATGAGTTTGAAGATTTGTTGGAGCATGTTAGTCCCCTTAAC

351 N L S S P I S M Y G S N E F E D L L E H V S P L N

TTTGGAGATCCCTTCAGAAAGCATGAGATGCGTTGCAGATTTAAGCAGAAACGCCCTTGGCCATGGCTAGCAATA

376 F G D P F R K H E M R C R F K Q K R P W P W L A I

ACAACTTCATATGGGATCCTTGTAATTACGCTTCTTGTTGCCCAAATATTTCATGCAACTATGAATAGAATAACC

401 T T S Y G I L V I T L L V A Q I F H A T M N R I T

AAGGTTGAAGATGATTTCCAAAAGATGAGAGAGCTGAAGAAACGTGCTGAGGCAGCTGATGTTGCCAAATCACAG

426 K V E D D F Q K M R E L K K R A E A A D V A K S Q

TTCCTTGCTACAGTTTCCCATGAGATCAGGACCCCAATGAATGGTGTGCTCGGGATGTTGCATATGCTCATGGAC

451 F L A T V S H E I R T P M N G V L G M L H M L M D

ACAGATCTTGATGTTACTCAACAAGACTATGTTAGGACTGCACAGGCCAGCGGAAAAGCTTTAGTCTCTCTTATA

476 T D L D V T Q Q D Y V R T A Q A S G K A L V S L I

AATGAGGTTCTGGACCAAGCAAAGATAGAAGCTGGTAAGCTAGAGCTCGAAGCAGTGCGGTTTGATCTGAGAGAG

501 N E V L D Q A K I E A G K L E L E A V R F D L R E

ATATTGGATGATGTTTTGTCTCTATTTTCTGGAAAATCTCAAGACAAGGGAGTGGAGTTGTCGGTTTACATTTCT

526 I L D D V L S L F S G K S Q D K G V E L S V Y I S

GACAAGGTCCCTGAGATGCTAATTGGCGATCCTGGAAGATTCCGACAGATCATCACAAATCTTATGGGCAACTCA

551 D K V P E M L I G D P G R F R Q I I T N L M G N S

ATCAAATTCACTGAGAAAGGGCATATCTTCGTAACGGTCCATCTTGTTGACGAGGTGATGGACATGACAGAAGTT

576 I K F T E K G H I F V T V H L V D E V M D M T E V

GAGAAGGAATCATCGTGGCAGAACACCTTAAGTGGGTTGCCAGTAGCAGATCAAAGCAAGAGCTGGGCAGGATTT

601 E K E S S W Q N T L S G L P V A D Q S K S W A G F

AAAATATTAAATCCAGAGGGGTCAAATTCTTCTGTATCATCTTCTTCTGATACCATCAATCTCATTGTATCAGTG

626 K I L N P E G S N S S V S S S S D T I N L I V S V

GAGGACACTGGTGTTGGAATCCCTTTAGAAGCCCAATCCCGTGTTTTCACTCCCTTTATGCAAGTAGGCCCCTCT

651 E D T G V G I P L E A Q S R V F T P F M Q V G P S

ATTTCTCGAACACATGGGGGCACAGGAATTGGATTAAGCATAAGCAAGTGCTTGGTTGGCCTTATGAATGGTGAA

676 I S R T H G G T G I G L S I S K C L V G L M N G E

ATCGGGTTTGTAAGCGTACCCAAGGTAGGATCCACCTTCACCTTTACTGCTGTATTTACGAATGGAGGCAATAGT

701 I G F V S V P K V G S T F T F T A V F T N G G N S

TCATATTCACAGAAGATTCAGCAAATCAATCAGCAGTCGGGATATAAAGAATTTCAAGGCATGAAAGCAATTGTT

726 S Y S Q K I Q Q I N Q Q S G Y K E F Q G M K A I V

GTGGACAGTAGGGAGGTCCGAGCAAAAGCATCAAGGTATCATATTCAACGTCTTGGGATCCAGGTCGAAGTAGTG

751 V D S R E V R A K A S R Y H I Q R L G I Q V E V V

ATTGGTTTGAGTCATGGTTTATCTGTCATGAGCAGTGGAAAAAAGGTTATTGACATGGTCCTAGTTGAACAAGAA

776 I G L S H G L S V M S S G K K V I D M V L V E Q E

GTTTGGGACAAGGATTTAGGCATGTCAGCTCTCTTTGTCAGTCGATTGATGAATTTTGACCAAGGTGTTCCTCCG

801 V W D K D L G M S A L F V S R L M N F D Q G V P P

AAAGTATTCCTTTTAGCTAACTCTGTAAATTCTAGCAGAAACGGGCATGCAACTTTGGGTAACTATTATCCATAC

826 K V F L L A N S V N S S R N G H A T L G N Y Y P Y

GTCATTGTGAAACCCTTGAGAGCAAGCATGCTGGCTGCTTCTATACAACGTGCCACGGGTGCAAAAAACCGAGGA

851 V I V K P L R A S M L A A S I Q R A T G A K N R G

AATTACCGAAATGGGGAGCTCCCGAGTTTGTCTCTCCGTAGTCTTCTCCTTGGTAGAAAAATTCTAGTTGTAGAT

876 N Y R N G E L P S L S L R S L L L G R K I L V V D

GACAATAATGTTAACCTCAGAGTAGCTGCTGGTGCATTGAAAAAGTATGGCGCTGACGTTGTCTGTGCAGACAGC

901 D N N V N L R V A A G A L K K Y G A D V V C A D S

GGAAAGAAGGCAATATCACTGCTGGAGCCCCCGCACCAGTTTGATGCTTGCTTCATGGATATCCAAATGCCTGAG

926 G K K A I S L L E P P H Q F D A C F M D I Q M P E

ATGGATGGGTTTGAAGCTACACGAAGGATCCGAGACATGGAATCTAAAATTAATGGCCGTATACAACATGGAGAA

951 M D G F E A T R R I R D M E S K I N G R I Q H G E

ATGTTAAAAGAAGCTCACAAAGACATCTCAAGATGTCATATTCCTATTATGGCAATGACTGCTGACGTGATTCAT

976 M L K E A H K D I S R C H I P I M A M T A D V I H

GCTACCAACGAAGAATGCCTAAAATCTGGAATGGATGGTTATGTCTCGAAACCGTTTGAAGCTGAACAACTCTAT

1001 A T N E E C L K S G M D G Y V S K P F E A E Q L Y

CGTGAGGTTTCACGCTTTTTCGTATAG

1026 R E V S R F F V *

**Fig. M** Nucleotide acid and deduced amino acid sequences of *DcHP1a* from carrot

ATGGCTGCTGTCATTCAGTTGCAAAGACAATTACTTGACTACACTACCTCCTTGTACAACGAGGCCTATTTGGAT

1 M A A V I Q L Q R Q L L D Y T T S L Y N E A Y L D

GGTCAGTTCACACAGCTTCAGCAGCTCCAAGATGAAAGCAACCCAGATTTTGTGGTTGAAGTTGTATCTCTTTTC

26 G Q F T Q L Q Q L Q D E S N P D F V V E V V S L F

TTCGAAGATTCTCAGAGGCTTCTTGATGATTTAACCGCTACTTTGAATCAGCAAGTTGTGGACTTTAAAAGGGTT

51 F E D S Q R L L D D L T A T L N Q Q V V D F K R V

GATGCCCATGTTCATCAGTTGAAAGGTAGCAGCTCCAGCATTGGAGCACAAAGAGTTCAGAGGGCTTGCATCATC

76 D A H V H Q L K G S S S S I G A Q R V Q R A C I I

TTCCGCAATTATTGCGAACAACAGAATACAGAAGGGTGTTTAAAATGCCTACAACAAGTGAAGAATGAGTATTAT

101 F R N Y C E Q Q N T E G C L K C L Q Q V K N E Y Y

TTGGTGAAGAACAAGCTTGAAACCCTGTTCAAGCTAGAGCAACAACTTGTGGCTGCTGGTGGATCAATTCCTATG

126 L V K N K L E T L F K L E Q Q L V A A G G S I P M

CAGTGA

151 Q *

**Fig. N** Nucleotide acid and deduced amino acid sequences of *DcHP1b* from carrot

ATGGAGTTGTTCCAAATGCAGAAGAGATATATTGAATACAATGCATCAATGTTTAGAGAGGGGTACTTTGATGAC

1 M E L F Q M Q K R Y I E Y N A S M F R E G Y F D D

CAGTTTAAACAGCTGCAACAACTGCAATATGGGGGCAGCCCTGGATTTGTTGCTGAAGTGGTTTCAATGTACCTC

26 Q F K Q L Q Q L Q Y G G S P G F V A E V V S M Y L

GCTGATTCGGATAGCCTTTTCAATGTTCTCACCGCAGCCATGGATGAGGAGCCTAAAGATTTCAAGAAGATGGAT

51 A D S D S L F N V L T A A M D E E P K D F K K M D

GGCTATGCTCATAAAATCAAGGGTAGCAGCGCCGGTGTCGGTGCTCAGCGAGTCCGAGACGCCTGCATTGCTTTT

76 G Y A H K I K G S S A G V G A Q R V R D A C I A F

CGTGGCTTCTGCGATGAACAGAACGCTGAAGAGTGCATGAGATGTCTGGTGCAAGCTAAACAAGAGTACGCAATT

101 R G F C D E Q N A E E C M R C L V Q A K Q E Y A I

GTGAAGGACAAGCTTCACCACTTGCTCGTGTTGGAGCGGCAGATTGTGGAAGCTGGTGGCATAGTTCCTCTGCTT

126 V K D K L H H L L V L E R Q I V E A G G I V P L L

CATTTGATGTGA

151 H L M *

**Fig. O** Nucleotide acid and deduced amino acid sequences of *DcHP3* from carrot

ATGGATATTGTCAATCAGTTGCAGCGACAGTATGTCGATTTCTTGTCTTCTCTTTATCGTGAGGGGCTTTTGGAT

1 M D I V N Q L Q R Q Y V D F L S S L Y R E G L L D

GATCAGTTCCTGCAGATTCAGAAGCTTCAGGATGAGAGTAACCCTGATTTTGTGAATGAAGTCGTTACTCTTTTC

26 D Q F L Q I Q K L Q D E S N P D F V N E V V T L F

TTTGAAGATTCAGAGAAGCTTTTGCACAACTTGGCCATAGCTCTGGACCAACAGCATGTGGATTATCAGAAAGTC

51 F E D S E K L L H N L A I A L D Q Q H V D Y Q K V

GATGCCTGTGTTCACCAATTCAAGGGTAGTAGCTCCAGCATAGGTGCTCAAAGAGTGAGGAACATATGTGTTTCT

76 D A C V H Q F K G S S S S I G A Q R V R N I C V S

TTCAGAACTTGTTATGATGCCAAGAACTTGGAAGGGTGCTTGAGATGCCTCCAGCAGGTGAAAGACGAATACTAC

101 F R T C Y D A K N L E G C L R C L Q Q V K D E Y Y

CTTGTTAAGAACAAGCTTCAGACATTGTTCAGGCTGGAGCAACAGATTGTAGCTGCTGGTGGAGCAATTCCGATT

126 L V K N K L Q T L F R L E Q Q I V A A G G A I P I

ATGGGATAG

151 M G *

**Fig. P** Nucleotide acid and deduced amino acid sequences of *DcRR-B1* from carrot

GCTACTACGTGCTCCCAGGCCACTGCTGCGTTAAACCTGCTACGGGAGAAAAAAGGTTGTTTTGATTTGGTACTG

1 A T T C S Q A T A A L N L L R E K K G C F D L V L

AGTGATGTACATATGCCTGATATGGACGGCTTCAAGCTCCTCGAACTTGTTGGTTTGGAAATGGACCTTCCTGTC

26 S D V H M P D M D G F K L L E L V G L E M D L P V

ATTATGATGTCAGCAGATGGAAGAACTAGTGCTGTTATGAGGGGAATTAAACATGGGGCTTGTGATTACTTAATC

51 I M M S A D G R T S A V M R G I K H G A C D Y L I

AAGCCTATACGTGAGGAAGAGTTAAAAAACATATGGCAACATGTTGTTCGGAAAAAGTGGAATGAAACTAAAGAA

76 K P I R E E E L K N I W Q H V V R K K W N E T K E

CAAGAGAATTCAAGCAGTTTGGATGAAAGTGATCGGCACCGCAGAGGAACCGATGATGTGGAATATGCTTCTTCT

101 Q E N S S S L D E S D R H R R G T D D V E Y A S S

GCTAATGAGGGATCAGAAGGAATATTAAAAGCTCAAAAAAAGAGGAGAGATGTTAAAGAAGAAGATGATGGTGAA

126 A N E G S E G I L K A Q K K R R D V K E E D D G E

TTCGATAATGATGACCCTTCTGCATCAAAGAAGCCACGTGTAGTCTGGTCGGTGGAGCTCCATCAGCAATTTGTT

151 F D N D D P S A S K K P R V V W S V E L H Q Q F V

AGTGCAGTAAACCAACTTGGAATAGAGAAGGCTGTTCCGAAAAGAATTCTCGAATTGATGAATGTCCCAGGCCTG

176 S A V N Q L G I E K A V P K R I L E L M N V P G L

ACTCGAGAAAATGTTGCAAGCCACTTGCAGAAATTCAGACTATATTTGAAGAGGTTAAGTGGTGTTTCACAACAG

201 T R E N V A S H L Q K F R L Y L K R L S G V S Q Q

CAAATTGGGCTTCCTAATTCTTTTTGTGGTCATATAGAGCAGAATCCGAAACTAGGCCCACTGGAAAGATTTGAT

226 Q I G L P N S F C G H I E Q N P K L G P L E R F D

TTCCAAGCTTTGGCTGCATCTGGTCAAATTCCTCCACAGACATTGGTAGCTCTGCATGCTGAGCTTTTAGGTCAA

251 F Q A L A A S G Q I P P Q T L V A L H A E L L G Q

CCCGCTGGAAATGTTGTATTACCAGCAATTCATCAGCAGTTTCTATTACAAGGATCTCGACAGGGACAGGAGAGT

276 P A G N V V L P A I H Q Q F L L Q G S R Q G Q E S

GTCCGTGGTGATGTAGGTGTGGCATATGGGCAGCCTCTGATAAAGTGCCCATCAAACATCTCCAAGCAATTTACC

301 V R G D V G V A Y G Q P L I K C P S N I S K Q F T

CAAGAATCGCCGGAAGATGTCAATTCAAATTTCAAGACATGGTCATCTAATAGCCTTGGTTCCATCGGTCCTAGC

326 Q E S P E D V N S N F K T W S S N S L G S I G P S

AGTAACCTTACAGGGCTAGCTTCACAGAACGGTAATACGTTAATGGGTGTTATGCAAAATCAGCAACAACTGCAA

351 S N L T G L A S Q N G N T L M G V M Q N Q Q Q L Q

AGGATGCTGCAAGAACAGCAGCATAAACAATCTGTTGTACAGGAATCCACCCCTTCCATCAATGTGCAACCTTCT

376 R M L Q E Q Q H K Q S V V Q E S T P S I N V Q P S

TGCCTTGTTGTCCCCGCTCAACCACCCAGTTTTCAGGTGGTAAATAGGCCTTCAATTAGTCAAAGCTGCAGCTTT

401 C L V V P A Q P P S F Q V V N R P S I S Q S C S F

CCTGGTGCTGAGGCTATCTTTAGTAGCGCTGATGTGACAACACCTCAGCCAAATAATTCCATATTTGCTGTTGGA

426 P G A E A I F S S A D V T T P Q P N N S I F A V G

CAAATGTTGGGTGTAAATCATAAAACTGCCAAGATTCTTGATGGATACTCAGTTGCCGGTCCTGTCTCTCCGTCT

451 Q M L G V N H K T A K I L D G Y S V A G P V S P S

GTGTCATCTTGCTCAGGAATTGCTGAAAATAATGCCTTGAGGAGAGTCCAAAATTCAGCCCCGGAATTTAATGCT

476 V S S C S G I A E N N A L R R V Q N S A P E F N A

TCTAGACAGGTGCCAGTTCTTTTTCGTGATATGAATGACTGCCAAGGTTCTTATAGTGAGAAATCTAGTCATGTG

501 S R Q V P V L F R D M N D C Q G S Y S E K S S H V

CTTGATCATGGAACACTGAGGAATATTGGATTTGTTGGCAAAGAAGCATGCATTCCTAGCCGTTTCGCAGTTGAT

526 L D H G T L R N I G F V G K E A C I P S R F A V D

GAAGTGGAAGCACCTATAAGCTGTAATGGAAGTACATATAGGGAAAATGGGAAAAAAGTGAAGCAGGAGCCAAAT

551 E V E A P I S C N G S T Y R E N G K K V K Q E P N

TTAGATTTCATCGAAAGTGTTAATATGAGTAACCCTATGATGCAATACTTCTCTCCGAATGATGTCATGAGTGTT

576 L D F I E S V N M S N P M M Q Y F S P N D V M S V

TTCTCCGAATAG

601 F S E *

**Fig. Q** Nucleotide acid and deduced amino acid sequences of *DcRR-B2* from carrot

ATGAATCTGGGTGGTGTTCAAGTAGCTAAATCTGGGTCTATGCCAAGTTCAAGCTCTCATGGGTCGACCCAGCAG

1 M N L G G V Q V A K S G S M P S S S S H G S T Q Q

TTTCCTGCGGGTCTTCGGGTCCTTGTGGTGGATGATGATCCAACTTGTCTCATGATCTTGGAGAAAATGCTCAGG

26 F P A G L R V L V V D D D P T C L M I L E K M L R

ACTTGTCACTATGAAGCGACAAAATGCAATCGAGCCGAGATTGCATTAAACCTACTAAGGGAGAACAGAAATGGA

51 T C H Y E A T K C N R A E I A L N L L R E N R N G

TATGATATTGTTTTAAGTGATGTTCACATGCCGGACATGGATGGGTTCAAGCTTCTTGAGCACATTGGACTGGAG

76 Y D I V L S D V H M P D M D G F K L L E H I G L E

ATGGACCTCCCTGTTATCATGATGTCTGCGGACGATAGCAAGAATGTTGTAATGAAAGGTGTAACTCATGGTGCC

101 M D L P V I M M S A D D S K N V V M K G V T H G A

TGTGATTACCTGATCAAACCAGTTCGTCTTGAAGCATTGAAGAACATATGGCAGCATGTAGTACGCAGAAAGAAA

126 C D Y L I K P V R L E A L K N I W Q H V V R R K K

CATGTGTGGAAGGACATTGAGCAATCCGGCAGTGTGGAAGATGGGAAGGTACAGCAACAGACACCTGACGATGCT

151 H V W K D I E Q S G S V E D G K V Q Q Q T P D D A

GATTACTTGTCTTCAGCAAATGAAGGCAGTTGGAGAAACTCAAAGAGAAGGAAGGATGAAGAAGAAGATGCAGAA

176 D Y L S S A N E G S W R N S K R R K D E E E D A E

GATAGAGATGATGACACATCGTCATTAAAGAAACCACGGGTTGTCTGGTCAGTTGAGCTTCACCAACAGTTTGTA

201 D R D D D T S S L K K P R V V W S V E L H Q Q F V

GCAGCCGCGAATCAACTTGGAATTGACAAGGCTGTTCCAAAGAAAATTCTAGAGTTGATGAATGTTCCTGGACTC

226 A A A N Q L G I D K A V P K K I L E L M N V P G L

ACTAGAGAAAATGTAGCCAGTCACCTTCAGAAATATCGCCTATATCTTAGAAGATTAAGCGGCCAGCACCAGAAT

251 T R E N V A S H L Q K Y R L Y L R R L S G Q H Q N

GCACTCAATGGCTCTTTCATGGGGAACCCAGAAGCAACTTATGGATCGATATCTTCGTTCAATGATCTAGAACTT

276 A L N G S F M G N P E A T Y G S I S S F N D L E L

CAAGCATATGCTGCAAGTGGTCAACTCCCAGCACAAAATCTTGCAGCACTTCAAGCAGCAGCGTTTGGCAGGTCT

301 Q A Y A A S G Q L P A Q N L A A L Q A A A F G R S

TCGTCGAAATCAGTTGTATCACCTCTAGATCAAAGAAACCTTTTTAGTTTTGAAACTCCAAAGTTACGCTTTGGA

326 S S K S V V S P L D Q R N L F S F E T P K L R F G

GAAGGGCAACAACAACACCTGAACAATAGTAAGCAGGTGAATTTCCTCCATGGTATCCCAACAAATATGGAGCCA

351 E G Q Q Q H L N N S K Q V N F L H G I P T N M E P

AAGCAACTTGCCAATTTTCATCAAGCTGCCAATTCATTTGGAATTAATATGCAAGTTGGCTCTCATGGAACCCAA

376 K Q L A N F H Q A A N S F G I N M Q V G S H G T Q

GGTAGTTCTCTGATGATGCAGATGGCTCAACCACAGTCAAGATCTCAAATACAAAATGACATTAATGGTAGTTAT

401 G S S L M M Q M A Q P Q S R S Q I Q N D I N G S Y

GTTTCAAGACTTCCACCATCCATCGGGCAGCCTGTTGTTTCAAATGTTGTTTCAAATGGGACAAGTAGAGTTTTG

426 V S R L P P S I G Q P V V S N V V S N G T S R V L

ACACGGCCAGTATATAATCATCAAGTCTCTCAAACCTCTCCAGTGGCAGGAATCTCAACACACCACTCTAATGAT

451 T R P V Y N H Q V S Q T S P V A G I S T H H S N D

CTGATGAGGAATAGCTTTCCTCTTGGGGGTAATTCTGGAATTTCATCTCTGGCTTCCAAAGTGATACCCCGAGAA

476 L M R N S F P L G G N S G I S S L A S K V I P R E

GAGGCACCTATCGAGATAAAAGGATCTCAAGGGGGTTCAAATTATGATGTATATAGTGGGCAGCAGCAGCGCAAA

501 E A P I E I K G S Q G G S N Y D V Y S G Q Q Q R K

TCTCAAGATTGGACCATACAGAATTCTGGATTGGCCTTCGACACCTTACAACATGCAAATATGCGAGGAAACCTT

526 S Q D W T I Q N S G L A F D T L Q H A N M R G N L

GATGTTTCACAAGCAGCCCTAGTTCAACAAAGCTTTTCATCTAGAGATGCAATTGAGCAGAGCAGGAATTCTTCT

551 D V S Q A A L V Q Q S F S S R D A I E Q S R N S S

GTTTTGCCATCTGTTTTACTTCAACAAGGTTTTCCTTCCAGTGGCCACAGCAGTAGCTCATCTTTTGGCAAGGGA

576 V L P S V L L Q Q G F P S S G H S S S S S F G K G

ATCTTCTCGGGAGTGGAAGAGAATGGACACAGGAATATGCCAAACACTGGTCAGCAGCTTAATGCTTATTTTACT

601 I F S G V E E N G H R N M P N T G Q Q L N A Y F T

GACACTTCACCAAGAGTCAAGGCTGAACTGCTTGAAGGAAATTTCTATAATAATTTACTGCCTGACCAATATGAT

626 D T S P R V K A E L L E G N F Y N N L L P D Q Y D

CAAGAAGACCTCATGTCTGCACTTCTAAAGCATCAGCAAGAAGGGATTGTAACAGTTGAAAATGATTTTGACTTC

651 Q E D L M S A L L K H Q Q E G I V T V E N D F D F

GACGGGTATCCCTTGGATAACCTTCCTGTCTAG

676 D G Y P L D N L P V *

**Fig. R** Nucleotide acid and deduced amino acid sequences of *DcRR-B3* from carrot

TTTGATAACAAGCACGATGATAAAAACTTTGGTGATCCTTCTGCTGGGAAGAGAACTAGAGTAGTTTGGACTGTC

1 F D N K H D D K N F G D P S A G K R T R V V W T V

GATCTTCATCAGAAGTTTGTCAAGGCCGTGAACTACATGGGATTTGACAAAGTTGGCCCCAAGAAAATACTCGAG

26 D L H Q K F V K A V N Y M G F D K V G P K K I L E

TTGATGAATGTGCCCTGGTTGACACGAGAAAATGTTGCTAGCCACTTGCAGAAGTACCGGTTATATTTGAGCCGA

51 L M N V P W L T R E N V A S H L Q K Y R L Y L S R

TTACAAAAAGATAATGATCTTAAAGCATCTCATGGTGGCATAAAGCAGTCCACTGATGCAACTTCAAGAGACCAA

76 L Q K D N D L K A S H G G I K Q S T D A T S R D Q

GCTGGAAACCTTGGCTTCAAGAATTCCATCAACATCAAACATAACGATGTTGCTAATACCAACTTCGGGATACCT

101 A G N L G F K N S I N I K H N D V A N T N F G I P

AAAAATACTGTTATCCAAAATTCCAACCCAAAAAGCCATGATGGTGTAGTTTCCTCACCGGATTTAGGCCCCAAG

126 K N T V I Q N S N P K S H D G V V S S P D L G P K

GGACCACTGGTTAGTGATGCTCATGAAATTCAGAAGGCAACCAGCAGTTCAGGAGTTGGCCTCCACTATAGTTTT

151 G P L V S D A H E I Q K A T S S S G V G L H Y S F

GGAACACCGGATCTAGATACCAAGTATACGACATTTTCTTCTGCCCTTCCACCTCAATTCTCTTGGAACCGAGAA

176 G T P D L D T K Y T T F S S A L P P Q F S W N R E

CATAAACCTCAGATTGATCCAAACAATGTTTTCAACCATCTGTCATTGCCTGATCTCGACCAAGTCCAAGTCAAT

201 H K P Q I D P N N V F N H L S L P D L D Q V Q V N

CAGAAAACTTTTCTTCCTAGCTATCCTACACCTTTCAACAAGGAGAGAGACAAACTGACACACATCAAAGCCATG

226 Q K T F L P S Y P T P F N K E R D K L T H I K A M

CCTCCGCGTGCCACAGAATGTATTAGTCTAAACATCAGACAACAAATGCCTGGAGAAAATACATTTGGATTAAAT

251 P P R A T E C I S L N I R Q Q M P G E N T F G L N

CCAGTTCAATCAGAGTGCTCTACGACAGCCTTTAATCCGTCTGAATCAATTACAAGA

276 P V Q S E C S T T A F N P S E S I T R

**Fig. S** Nucleotide acid and deduced amino acid sequences of *DcRR-B4* from carrot

ATGACTGTTGAGGAAACAAGAGCAAGTTCTGGGAATGATTTTGATGATTTTCCAAAGGGTATGCGTGTTCTTGCT

1 M T V E E T R A S S G N D F D D F P K G M R V L A

GTTGATGATGACCCTACTTGTTTGAAGTTGTTAGAAGGTCTCCTCAGGAAATGCCAGTATCATGTTACACTTGCA

26 V D D D P T C L K L L E G L L R K C Q Y H V T L A

AATCAGGCAAGAATAGCATTGAATATGCTTCGAGAAAACAGAAACAGATTTGACCTGATTATTAGTGATGTGCAT

51 N Q A R I A L N M L R E N R N R F D L I I S D V H

ATGCCGGACATGGATGGTTTTAAGCTACTGGAGCTTGTTGGACTTGAAATGGACCTTCCTGTTATTATGTTGTCG

76 M P D M D G F K L L E L V G L E M D L P V I M L S

GCAAACAGTGATCCTAAACTAGTAATGAAGGGGATTACTCATGGTGCTTGTGACTATCTTGTAAAACCTGTTCGA

101 A N S D P K L V M K G I T H G A C D Y L V K P V R

GTTGAGGAACTCAGAAACATATGGCAACATGTGGTCCGAAAGAAGGTTGACCCTAAGTCACGGGACAAGTCCAAC

126 V E E L R N I W Q H V V R K K V D P K S R D K S N

TCTTCTTACGATAGAGATCAGCAGGGAAGTGAAGGTGGACAAGGACCTGCTGTAACTGGTAATGCAGACCAGGAT

151 S S Y D R D Q Q G S E G G Q G P A V T G N A D Q D

GGAAAATCGAGTAGGAAACGTAAGGATGATGAAGAAGAAGATGAAGAGAATGACCAGGATGATGTCGACCCATCA

176 G K S S R K R K D D E E E D E E N D Q D D V D P S

GCACAGAAGAAACCTCGGGTAGTATGGTCTATAGAGCTTCATCGGAAATTTGTAGCAGCTGTTAATCAGCTAGGC

201 A Q K K P R V V W S I E L H R K F V A A V N Q L G

CTTGAAAAAGCTGTTCCAAAAAGAATTCTTGATCTTATGAACGTTGATGGGCTCACAAGGGAAAACGTAGCTAGC

226 L E K A V P K R I L D L M N V D G L T R E N V A S

CATCTTCAGAAATACCGACTGTACCTGAAAAGGATAAGTACAGCCGCATCTCAGCAAGCTAATATGGTTGCTGCA

251 H L Q K Y R L Y L K R I S T A A S Q Q A N M V A A

CTAGGTGGTAAGGATGCCTCCTATTTGCGCATGGGATCTCTTGATGGATATGGAGATTATCGAACTTTAACTGGA

276 L G G K D A S Y L R M G S L D G Y G D Y R T L T G

TCAGGAAGGCTTCCAGGCACCGGGTTGTCCTCTTATGCACCCTCTGGAATGCTCGGAAGATTGAATAGTCCTGCT

301 S G R L P G T G L S S Y A P S G M L G R L N S P A

GGAGTGAGCCTCCGTGGCCTTGCTTCCCCTACTATTATCCAGCCAAATCTTGCACAAAGCCTAAGCAACCCTGCT

326 G V S L R G L A S P T I I Q P N L A Q S L S N P A

TTTGGGAAGTTTCAGACAATTGGTTCGCAATCCCACCAAAACCCAAACTTCTTTCAAGGAATTCCATCGTCCTTG

351 F G K F Q T I G S Q S H Q N P N F F Q G I P S S L

GAGCTTGATCAGTTCCAGCAGACTAAGTCTACTACTCAGGTTGGAGACTTTAGTGCCATAGACAGCCACAGAAGT

376 E L D Q F Q Q T K S T T Q V G D F S A I D S H R S

TTTTCTGCTGCTACTAATAGCTTTGCGGACACGCGAGCTGCAGTGAATTCAAGCAATTTACTAGGTACGGGTCCA

401 F S A A T N S F A D T R A A V N S S N L L G T G P

AGCAACCCTTTACTTTTACAGGGCAACACACAGCAGATACAGAATGGTCTCGGATTTGTTAATCAGTCCTCTGTC

426 S N P L L L Q G N T Q Q I Q N G L G F V N Q S S V

AACGTTCCTTCTTTGACCTCGGATTCTTTTAATGTCGGTACCAGTGGTTCATCTAATATCATGGATGATGGCAGA

451 N V P S L T S D S F N V G T S G S S N I M D D G R

GGTCAAGAGTGGCCCTTTCAAGTATCAAAGTTTTCATCAAACCCTTTACCGTTAAATGAACCCTTCAATACTGCT

476 G Q E W P F Q V S K F S S N P L P L N E P F N T A

CAGATGCCTCCTAACAGCATGATATACAATAGTTCTTCTTCAAGCATACATATTCAGAACTCCCCAATGCAATTT

501 Q M P P N S M I Y N S S S S S I H I Q N S P M Q F

CCCTCTAATTCTACAATATCTGCACCTATTGAAGATTCAAGAGGAGAACACCAAGGCGGGTTGGTTGGTAATTTT

526 P S N S T I S A P I E D S R G E H Q G G L V G N F

GTTCAGAATGTAAACCAATTACCCAATCAAAGATTTAGGCAACATAAACAAGATTACACAAACAACTCAAATAAT

551 V Q N V N Q L P N Q R F R Q H K Q D Y T N N S N N

GCTTTTAGCGCTCTGAGCACACTTTCTAATTCTAATGGTGGAACGACTCCCTTTAGCCAGACTGTGGACCAAGAT

576 A F S A L S T L S N S N G G T T P F S Q T V D Q D

AATGGGGGCTACAATCGAAGGGTGAATGGAAGTGTTTCTAGTATCATGCAACATGGTGAAACTGAAAAATCACCT

601 N G G Y N R R V N G S V S S I M Q H G E T E K S P

ATGAACATGAAAATGAGGTCCACTGATGGCTACCTTTTGGATCAAACAAAGTCGCATGGTGGTTTGGTTCCTAAT

626 M N M K M R S T D G Y L L D Q T K S H G G L V P N

AATTATGATTCGTTGGATGATATAATGAATGCTGTAATGAAACGGGAGCAAGACGGGGCAATGTTAATGGAGGGA

651 N Y D S L D D I M N A V M K R E Q D G A M L M E G

GAATTCGGACTTGATGCTTACACTTTCGGATCATGTATATGA

676 E F G L D A Y T F G S C I *

**Fig. T** Nucleotide acid and deduced amino acid sequences of *DcRR-A1* from carrot

ATGGGCATGGCCACTACTAAGTCTGAGTTTCATGTTTTGGCTGTTGATGATAGCATCATAGATAGGAAACTCATT

1 M G M A T T K S E F H V L A V D D S I I D R K L I

GAGAGGCTTCTCAAAACTGCTTCATATCAAGTTACAACAGTTGATTCTGGGAGCAAAGCTCTGGAGTTTCTGGGT

26 E R L L K T A S Y Q V T T V D S G S K A L E F L G

TTTTGTCAAGATGTGGAAAGCAACTCAAATGAACCTTCCATCTCCCCGAATAATCAGCAGGAGGTGGAAGTGAAC

51 F C Q D V E S N S N E P S I S P N N Q Q E V E V N

CTAATAATTACAGACTACTGTATGCCAGAGATGACAGGCTATGATTTGCTGAAGAAAATTAAGGAATCTTCATCT

76 L I I T D Y C M P E M T G Y D L L K K I K E S S S

TTCAGAGACATACCAGTAGTGATCATGTCCTCTGAAAATGTTCCTTCAAGAATCAGCAGATGTTTGGAAGAAGGA

101 F R D I P V V I M S S E N V P S R I S R C L E E G

GCCGAAGAATTTTTTCTGAAACCAGTAAGATTAGCCGATGTGAATAAGCTAAAACCCCATATGATGAAAACCAAA

126 A E E F F L K P V R L A D V N K L K P H M M K T K

AACAGAGATTGTCAAAAGGCAGAACAGGAGGAAGTATCATCACAGGAGAAAATAGTCCCGCCAGAGGTTCAGTCT

151 N R D C Q K A E Q E E V S S Q E K I V P P E V Q S

TCAGAACCACCACAGCCAGATGACGGCAATAACACGAAGAGAAAGTCTACGGAAGTGGAAGAAGAAGTTTCACAA

176 S E P P Q P D D G N N T K R K S T E V E E E V S Q

GACAGAACCCGGCGGAGATACAACGGGTTCACAGTAATTTGA

201 D R T R R R Y N G F T V I *

**Fig. U** Nucleotide acid and deduced amino acid sequences of *DcRR-A2* from carrot

GACTACTGTATGCCTGGAATGACAGGCTATGATTTGCTCAAGAGAATAAAGGAGTCATCTTTCAGAGACATTCCA

1 D Y C M P G M T G Y D L L K R I K E S S F R D I P

GTTGTGATTATGTCATCTGAGAATGTCCCTTCAAGAATTAATAGATGTTTAGAGGAAGGAGCAGATGAGTTTTTT

26 V V I M S S E N V P S R I N R C L E E G A D E F F

CTTAAACCAGTGAGATTATCAGATGTGAACAAGCTTAAACCCCATATAATGAAAAGAAAGAATTTAGATGGTGAG

51 L K P V R L S D V N K L K P H I M K R K N L D G E

AATCTGAAAAGGGAAACAGGTCAAGAACAAGCCTTGGAGGGTTTAGAAGTGATTCAGTCATTACAGCAAGAAGCA

76 N L K R E T G Q E Q A L E G L E V I Q S L Q Q E A

GTAGCCCCACAAAAACAAGGCATTGGTAATAATAACAAGAGGAAGGCTATGGAATTGGAAGAAGCACTCTCACAA

101 V A P Q K Q G I G N N N K R K A M E L E E A L S Q

GATCATAGAACAAGACCAAGATGCAGTGGCCTCACTGTCATTTAG

126 D H R T R P R C S G L T V I *

**Table A.** Raw Cq (quantification cycle) values of genes in different tissues during carrot growth and development.

| **Tissue types** | **Stages** | ***DcIPT3*** | | ***DcIPT5*** | | ***DcIPT9*** | | ***DcCYP235A1*** | | ***DcCYP235A2*** | | ***DcLOG1*** | |
| --- | --- | --- | --- | --- | --- | --- | --- | --- | --- | --- | --- | --- | --- |
| Root | 1 | 25.17 | | 26.42 | | 27.33 | | 27.50 | | 27.21 | | 25.84 | |
|  |  | 24.71 | | 26.61 | | 26.74 | | 27.08 | | 27.14 | | 26.00 | |
|  |  | 24.24 | | 26.22 | | 26.12 | | 27.17 | | 27.73 | | 24.65 | |
|  | 2 | 26.99 | | 30.84 | | 25.33 | | 27.73 | | 24.94 | | 25.95 | |
|  |  | 26.74 | | 30.83 | | 25.65 | | 27.98 | | 25.36 | | 26.16 | |
|  |  | 27.79 | | N/A | | 26.04 | | 26.57 | | 25.71 | | 25.78 | |
|  | 3 | 28.17 | | 31.00 | | 26.32 | | 28.61 | | 28.63 | | 27.90 | |
|  |  | 28.57 | | 30.55 | | 26.29 | | 24.32 | | 28.65 | | 27.19 | |
|  |  | 28.92 | | 34.45 | | 27.13 | | 28.85 | | 27.92 | | 27.71 | |
|  | 4 | 27.59 | | 31.76 | | 25.68 | | 27.16 | | 28.58 | | 30.18 | |
|  |  | 27.59 | | 31.24 | | 25.50 | | 26.52 | | 28.22 | | 30.70 | |
|  |  | 27.31 | | 31.05 | | 24.64 | | 26.85 | | 29.24 | | 30.13 | |
|  | 5 | 28.05 | | 30.64 | | 26.39 | | 27.56 | | 29.18 | | 31.30 | |
|  |  | 28.23 | | 31.17 | | 26.11 | | 27.01 | | 28.65 | | 31.73 | |
|  |  | 28.52 | | 29.71 | | 26.00 | | 26.71 | | 28.51 | | 32.02 | |
| Petiole | 1 | 28.24 | | 33.33 | | 28.58 | | 28.50 | | 31.76 | | 27.35 | |
|  |  | 28.69 | | N/A | | 28.16 | | 28.81 | | 31.63 | | 27.09 | |
|  |  | 28.92 | | 32.83 | | 29.05 | | 28.86 | | 31.61 | | 27.08 | |
|  | 2 | 30.30 | | 31.60 | | 27.21 | | 27.50 | | 28.76 | | 24.75 | |
|  |  | 30.13 | | 31.89 | | 27.24 | | 26.83 | | 29.06 | | 24.61 | |
|  |  | 30.33 | | 30.86 | | 26.70 | | 26.26 | | 28.54 | | 24.27 | |
|  | 3 | 31.62 | | 38.20 | | 28.77 | | 29.75 | | 32.96 | | 28.25 | |
|  |  | 31.90 | | 34.89 | | 28.95 | | 28.80 | | 31.63 | | 28.35 | |
|  |  | 34.07 | | 34.65 | | 28.58 | | 28.29 | | 32.01 | | 28.55 | |
|  | 4 | 30.44 | | 35.61 | | 28.10 | | 27.54 | | 30.85 | | 26.00 | |
|  |  | 30.02 | | 34.78 | | 27.14 | | 27.80 | | 29.70 | | 26.01 | |
|  |  | 28.90 | | 34.55 | | 26.97 | | 27.15 | | 29.55 | | 25.74 | |
|  | 5 | 27.67 | | 29.06 | | 27.34 | | 27.93 | | 31.38 | | 26.74 | |
|  |  | 28.92 | | 29.12 | | 28.09 | | 27.81 | | 31.22 | | 26.94 | |
|  |  | 29.55 | | 29.66 | | 28.37 | | 27.87 | | 33.66 | | 27.19 | |
| Leaf | 1 | 25.24 | | 33.46 | | 26.77 | | 33.73 | | 30.80 | | 25.75 | |
|  |  | 25.27 | | 33.63 | | 26.63 | | 33.27 | | 30.96 | | 25.68 | |
|  |  | 24.80 | | 32.71 | | 26.86 | | 33.09 | | 30.86 | | 25.28 | |
|  | 2 | 27.23 | | 34.00 | | 25.40 | | 32.54 | | 28.27 | | 23.28 | |
|  |  | 27.55 | | 32.67 | | 25.61 | | 31.86 | | 28.63 | | 23.89 | |
|  |  | 26.17 | | 33.11 | | 23.78 | | 31.67 | | 29.62 | | 23.19 | |
|  | 3 | 29.27 | | 35.18 | | 31.18 | | 34.30 | | 35.74 | | 29.89 | |
|  |  | 29.61 | | 34.59 | | 30.42 | | 32.73 | | 35.65 | | 29.34 | |
|  |  | 29.65 | | N/A | | 29.78 | | 33.40 | | N/A | | 28.87 | |
|  | 4 | 27.63 | | 33.38 | | 29.86 | | 36.26 | | 33.39 | | 27.07 | |
|  |  | 27.78 | | N/A | | 29.68 | | 34.33 | | 33.73 | | 27.06 | |
|  |  | 27.64 | | 34.13 | | 30.10 | | 33.83 | | .65 | | 27.99 | |
|  | 5 | 25.77 | | 34.40 | | 27.49 | | 34.19 | | 32.58 | | 29.43 | |
|  |  | 26.64 | | 34.40 | | 27.06 | | 31.77 | | 32.80 | | 29.42 | |
|  |  | 25.70 | | 34.44 | | 28.65 | | 33.82 | | 33.79 | | 29.55 | |
| **Tissue types** | **Stages** | ***DcLOG3*** | ***DcLOG8*** | | ***DcCYX1*** | | ***DcCYX7*** | | ***DcHK2*** | | ***DcHK3*** | |  |
| Root | 1 | 24.03 | 22.41 | | 25.97 | | 26.12 | | 25.55 | | 27.10 | |  |
|  |  | 23.61 | 22.56 | | 26.69 | | 26.64 | | 25.75 | | 27.12 | |  |
|  |  | 23.30 | 20.67 | | 24.94 | | 25.31 | | 25.83 | | 26.83 | |  |
|  | 2 | 22.78 | 22.61 | | 25.48 | | 28.20 | | 24.47 | | 26.27 | |  |
|  |  | 24.18 | 21.83 | | 25.19 | | 28.31 | | 24.03 | | 26.46 | |  |
|  |  | 24.62 | 22.78 | | 25.80 | | 28.17 | | 24.96 | | 25.99 | |  |
|  | 3 | 22.81 | 24.62 | | 29.56 | | 26.64 | | 25.80 | | 26.44 | |  |
|  |  | 22.18 | 24.46 | | 29.87 | | 27.73 | | 25.62 | | 26.40 | |  |
|  |  | 22.66 | 24.24 | | 28.84 | | 26.99 | | 26.68 | | 25.48 | |  |
|  | 4 | 23.49 | 23.65 | | 28.33 | | 30.12 | | 26.27 | | 27.13 | |  |
|  |  | 24.03 | 23.88 | | 28.18 | | 30.63 | | 25.42 | | 26.68 | |  |
|  |  | 24.65 | 24.05 | | 28.04 | | 29.95 | | 25.77 | | 26.65 | |  |
|  | 5 | 24.52 | 23.21 | | 31.84 | | 24.74 | | 27.00 | | 28.33 | |  |
|  |  | 23.67 | 23.68 | | 32.23 | | 24.20 | | 27.29 | | 27.85 | |  |
|  |  | 24.51 | 23.47 | | 32.62 | | 23.96 | | 27.60 | | 27.11 | |  |
| Petiole | 1 | 28.19 | 24.49 | | 28.71 | | 27.35 | | 27.67 | | 28.37 | |  |
|  |  | 27.59 | 25.05 | | 28.73 | | 27.08 | | 27.82 | | 28.42 | |  |
|  |  | 28.06 | 25.16 | | 28.75 | | 28.19 | | 27.84 | | 28.09 | |  |
|  | 2 | 26.54 | 21.87 | | 27.43 | | 25.66 | | 25.99 | | 25.91 | |  |
|  |  | 25.99 | 21.89 | | 27.41 | | 25.63 | | 26.03 | | 25.88 | |  |
|  |  | 22.89 | 21.88 | | 27.40 | | 25.48 | | 25.82 | | 25.69 | |  |
|  | 3 | 27.12 | 24.45 | | 27.86 | | 25.82 | | 27.48 | | 27.82 | |  |
|  |  | 28.58 | 23.74 | | 27.93 | | 25.95 | | 27.66 | | 27.24 | |  |
|  |  | 28.55 | 25.13 | | 28.59 | | 26.44 | | 27.09 | | 26.73 | |  |
|  | 4 | 25.81 | 23.75 | | 28.16 | | 26.30 | | 26.43 | | 25.67 | |  |
|  |  | 25.09 | 23.05 | | 28.25 | | 25.72 | | 25.78 | | 26.08 | |  |
|  |  | 24.23 | 22.98 | | 27.14 | | 25.96 | | 25.16 | | 25.06 | |  |
|  | 5 | 25.45 | 23.26 | | 25.91 | | 26.85 | | 25.93 | | 26.47 | |  |
|  |  | 25.26 | 23.21 | | 25.74 | | 27.23 | | 25.77 | | 26.64 | |  |
|  |  | 25.00 | 23.73 | | 25.98 | | 27.24 | | 26.11 | | 26.63 | |  |
| Leaf | 1 | 27.61 | 24.99 | | 32.85 | | 24.62 | | 28.20 | | 26.52 | |  |
|  |  | 26.35 | 24.98 | | 32.86 | | 24.71 | | 28.13 | | 26.54 | |  |
|  |  | 26.00 | 24.17 | | 32.69 | | 24.77 | | 27.63 | | 26.25 | |  |
|  | 2 | 25.68 | 22.56 | | 33.74 | | 22.66 | | 26.74 | | 26.39 | |  |
|  |  | 23.83 | 23.35 | | 35.05 | | 23.70 | | 27.15 | | 26.60 | |  |
|  |  | 25.89 | 23.93 | | 33.65 | | 23.83 | | 26.01 | | 27.06 | |  |
|  | 3 | 31.11 | 26.74 | | 34.01 | | 25.84 | | 30.96 | | 29.50 | |  |
|  |  | 31.27 | 25.67 | | 33.45 | | 25.58 | | 31.40 | | 28.96 | |  |
|  |  | 31.68 | 25.60 | | 34.84 | | 24.24 | | 31.72 | | 28.46 | |  |
|  | 4 | 28.73 | 26.08 | | 32.60 | | 23.74 | | 31.39 | | 28.30 | |  |
|  |  | 28.70 | 26.13 | | 34.01 | | 23.74 | | 31.43 | | 27.81 | |  |
|  |  | 28.61 | 26.46 | | 32.66 | | 23.82 | | 30.41 | | 28.82 | |  |
|  | 5 | 27.86 | 25.18 | | 33.64 | | 21.72 | | 31.72 | | 28.71 | |  |
|  |  | 27.79 | 24.69 | | 33.59 | | 21.53 | | 30.84 | | 28.62 | |  |
|  |  | 27.77 | 24.29 | | 34.74 | | 21.59 | | 30.24 | | 29.02 | |  |
| **Tissue types** | **Stages** | ***DcHP1a*** | ***DcHP1b*** | | ***DcHP3*** | | ***DcRR-B1*** | | ***DcRR-B2*** | | ***DcRR-B3*** | |  |
| Root | 1 | 21.71 | 23.12 | | 22.63 | | 25.23 | | 24.41 | | 23.98 | |  |
|  |  | 21.94 | 22.59 | | 22.10 | | 25.36 | | 24.91 | | 24.75 | |  |
|  |  | 21.62 | 22.75 | | 21.92 | | 24.63 | | 23.52 | | 24.99 | |  |
|  | 2 | 21.14 | 22.24 | | 22.36 | | 24.44 | | 24.62 | | 25.93 | |  |
|  |  | 21.14 | 22.33 | | 21.85 | | 24.20 | | 24.43 | | 26.12 | |  |
|  |  | 22.07 | 22.94 | | 21.38 | | 23.78 | | 24.98 | | 26.70 | |  |
|  | 3 | 22.90 | 24.90 | | 22.21 | | 24.34 | | 25.69 | | 27.72 | |  |
|  |  | 22.51 | 25.11 | | 22.86 | | 23.82 | | 26.16 | | 28.11 | |  |
|  |  | 23.24 | 25.55 | | 21.51 | | 23.69 | | 25.97 | | 28.48 | |  |
|  | 4 | 22.11 | 24.68 | | 22.06 | | 24.33 | | 30.54 | | 27.90 | |  |
|  |  | 21.97 | 24.54 | | 22.49 | | 24.83 | | 25.28 | | 27.55 | |  |
|  |  | 20.54 | 24.27 | | 22.21 | | 24.98 | | 25.11 | | 25.62 | |  |
|  | 5 | 23.79 | 24.40 | | 23.25 | | 26.65 | | 26.73 | | 30.82 | |  |
|  |  | 23.79 | 25.33 | | 22.69 | | 26.93 | | 26.91 | | 30.81 | |  |
|  |  | 23.10 | 24.81 | | 22.34 | | 25.79 | | 26.97 | | 30.97 | |  |
| Petiole | 1 | 25.21 | 27.74 | | 25.81 | | 27.46 | | 27.48 | | 29.66 | |  |
|  |  | 25.07 | 28.54 | | 25.67 | | 27.75 | | 27.33 | | 30.06 | |  |
|  |  | 25.55 | 28.51 | | 25.39 | | 27.20 | | 27.46 | | 31.46 | |  |
|  | 2 | 22.81 | 26.46 | | 22.22 | | 25.70 | | 25.37 | | 26.45 | |  |
|  |  | 22.82 | 26.54 | | 22.22 | | 25.55 | | 24.73 | | 26.40 | |  |
|  |  | 22.24 | 26.48 | | 22.72 | | 25.60 | | 25.05 | | 25.62 | |  |
|  | 3 | 26.26 | 28.95 | | 25.72 | | 27.73 | | 27.73 | | 30.60 | |  |
|  |  | 26.45 | 29.39 | | 25.60 | | 27.09 | | 27.34 | | 29.59 | |  |
|  |  | 25.60 | 29.31 | | 25.77 | | 26.73 | | 26.90 | | 28.91 | |  |
|  | 4 | 24.63 | 28.82 | | 23.50 | | 24.81 | | 24.52 | | 27.73 | |  |
|  |  | 23.91 | 27.49 | | 23.95 | | 25.01 | | 24.68 | | 26.93 | |  |
|  |  | 23.67 | 27.24 | | 23.25 | | 24.03 | | 23.93 | | 27.29 | |  |
|  | 5 | 25.08 | 26.85 | | 23.25 | | 26.17 | | 25.86 | | 26.07 | |  |
|  |  | 24.94 | 27.49 | | 23.15 | | 26.63 | | 25.87 | | 26.47 | |  |
|  |  | 25.21 | 27.83 | | 23.55 | | 26.53 | | 26.20 | | 26.48 | |  |
| Leaf | 1 | 24.57 | 28.62 | | 25.32 | | 26.30 | | 26.63 | | 35.09 | |  |
|  |  | 24.66 | 28.42 | | 24.99 | | 26.63 | | 26.73 | | N/A | |  |
|  |  | 24.15 | 27.65 | | 24.69 | | 26.53 | | 26.75 | | 34.75 | |  |
|  | 2 | 22.72 | 26.98 | | 23.48 | | 25.95 | | 26.24 | | 33.35 | |  |
|  |  | 23.24 | 27.24 | | 24.39 | | 26.59 | | 26.66 | | 32.73 | |  |
|  |  | 22.22 | 27.22 | | 24.52 | | 26.48 | | 25.85 | | 31.65 | |  |
|  | 3 | 26.56 | 29.48 | | 27.95 | | 30.20 | | 29.57 | | 31.88 | |  |
|  |  | 26.93 | 29.37 | | 27.14 | | 29.94 | | 29.60 | | 32.56 | |  |
|  |  | 26.08 | 30.37 | | 26.78 | | 29.81 | | 28.34 | | 33.27 | |  |
|  | 4 | 26.76 | 28.70 | | 23.85 | | 28.39 | | 26.34 | | 34.68 | |  |
|  |  | 26.46 | 29.09 | | 23.84 | | 27.59 | | 26.61 | | 30.82 | |  |
|  |  | 26.85 | 28.17 | | 24.66 | | 28.70 | | 27.67 | | 35.21 | |  |
|  | 5 | 26.40 | 28.20 | | 24.17 | | 28.29 | | 28.72 | | 35.22 | |  |
|  |  | 26.51 | 28.45 | | 24.54 | | 28.05 | | 29.09 | | 35.87 | |  |
|  |  | 25.51 | 28.59 | | 24.81 | | 29.30 | | 29.65 | | 35.91 | |  |
| **Tissue types** | **Stages** | ***DcRR-B4*** | ***DcRR-A1*** | | ***DcRR-A2*** | | ***DcACTIN*** | |  |  |  |  |  |
| Root | 1 | 24.53 | 22.78 | | 23.27 | | 19.43 | |  |  |  |  |  |
|  |  | 24.26 | 22.78 | | 23.27 | | 19.92 | |  |  |  |  |  |
|  |  | 24.12 | 22.34 | | 19.80 | | 19.27 | |  |  |  |  |  |
|  | 2 | 24.66 | 21.62 | | 22.74 | | 19.57 | |  |  |  |  |  |
|  |  | 24.30 | 21.67 | | 21.52 | | 19.19 | |  |  |  |  |  |
|  |  | 25.30 | 22.39 | | 23.11 | | 19.86 | |  |  |  |  |  |
|  | 3 | 26.29 | 25.48 | | 25.18 | | 19.91 | |  |  |  |  |  |
|  |  | 26.11 | 25.62 | | 7.82 | | 19.00 | |  |  |  |  |  |
|  |  | 26.92 | 25.83 | | 25.14 | | 20.30 | |  |  |  |  |  |
|  | 4 | 25.19 | 23.99 | | 24.61 | | 19.04 | |  |  |  |  |  |
|  |  | 25.09 | 24.07 | | 24.74 | | 19.59 | |  |  |  |  |  |
|  |  | 23.59 | 22.25 | | 25.19 | | 20.58 | |  |  |  |  |  |
|  | 5 | 27.95 | 25.70 | | 23.82 | | 18.59 | |  |  |  |  |  |
|  |  | 27.54 | 25.48 | | 23.08 | | 19.71 | |  |  |  |  |  |
|  |  | 26.82 | 25.20 | | 23.08 | | 19.53 | |  |  |  |  |  |
| Petiole | 1 | 26.79 | 25.95 | | 24.73 | | 21.82 | |  |  |  |  |  |
|  |  | 27.00 | 26.49 | | 24.82 | | 22.03 | |  |  |  |  |  |
|  |  | 27.28 | 26.24 | | 24.72 | | 21.85 | |  |  |  |  |  |
|  | 2 | 25.57 | 25.20 | | 24.99 | | 19.79 | |  |  |  |  |  |
|  |  | 25.44 | 25.21 | | 24.49 | | 19.86 | |  |  |  |  |  |
|  |  | 25.66 | 24.84 | | 24.71 | | 19.79 | |  |  |  |  |  |
|  | 3 | 28.37 | 29.99 | | 28.41 | | 20.91 | |  |  |  |  |  |
|  |  | 27.73 | 29.63 | | 28.43 | | 22.99 | |  |  |  |  |  |
|  |  | 28.69 | 26.77 | | 28.36 | | 23.09 | |  |  |  |  |  |
|  | 4 | 25.90 | 26.29 | | 25.39 | | 19.88 | |  |  |  |  |  |
|  |  | 25.53 | 26.37 | | 25.74 | | 20.62 | |  |  |  |  |  |
|  |  | 25.07 | 26.15 | | 25.12 | | 19.72 | |  |  |  |  |  |
|  | 5 | 26.16 | 27.41 | | 26.97 | | 21.04 | |  |  |  |  |  |
|  |  | 25.71 | 27.95 | | 27.40 | | 21.21 | |  |  |  |  |  |
|  |  | 26.23 | 28.69 | | 27.29 | | 21.32 | |  |  |  |  |  |
| Leaf | 1 | 27.82 | 28.25 | | 26.60 | | 22.55 | |  |  |  |  |  |
|  |  | 27.28 | 28.34 | | 26.45 | | 22.76 | |  |  |  |  |  |
|  |  | 27.37 | 27.84 | | 25.87 | | 22.45 | |  |  |  |  |  |
|  | 2 | 25.99 | 27.42 | | 26.36 | | 20.80 | |  |  |  |  |  |
|  |  | 26.70 | 26.99 | | 27.03 | | 21.50 | |  |  |  |  |  |
|  |  | 24.80 | 26.97 | | 29.64 | | 21.49 | |  |  |  |  |  |
|  | 3 | 30.31 | 31.42 | | 30.24 | | 24.90 | |  |  |  |  |  |
|  |  | 29.79 | 31.40 | | 30.92 | | 24.75 | |  |  |  |  |  |
|  |  | 28.20 | 31.60 | | 30.66 | | 24.32 | |  |  |  |  |  |
|  | 4 | 29.05 | 29.34 | | 26.87 | | 22.73 | |  |  |  |  |  |
|  |  | 28.26 | 28.52 | | 26.66 | | 22.58 | |  |  |  |  |  |
|  |  | 28.88 | 28.86 | | 27.63 | | 23.84 | |  |  |  |  |  |
|  | 5 | 28.76 | 28.19 | | 25.34 | | 22.61 | |  |  |  |  |  |
|  |  | 29.15 | 28.96 | | 25.06 | | 22.73 | |  |  |  |  |  |
|  |  | 28.90 | 28.75 | | 26.02 | | 23.44 | |  |  |  |  |  |
